# Odorant mixtures elicit less variable and faster responses than pure odorants

**DOI:** 10.1371/journal.pcbi.1006536

**Published:** 2018-12-10

**Authors:** Ho Ka Chan, Fabian Hersperger, Emiliano Marachlian, Brian H. Smith, Fernando Locatelli, Paul Szyszka, Thomas Nowotny

**Affiliations:** 1 Sussex Neuroscience, School of Engineering and Informatics, University of Sussex, Falmer, Brighton, United Kingdom; 2 Department of Neuroscience, University of Konstanz, Konstanz, Germany; 3 Instituto de Fisiología, Biología Molecular y Neurociencias (IFIBYNE-UBA-CONICET) and Departamento de Fisiología, Biología Molecular y Celular, Facultad de Ciencias Exactas y Naturales, Universidad de Buenos Aires, Ciudad Universitaria, Buenos Aires, Argentina; 4 School of Life Sciences, Arizona State University, Tempe, Arizona, United States of America; University College London, UNITED KINGDOM

## Abstract

In natural environments, odors are typically mixtures of several different chemical compounds. However, the implications of mixtures for odor processing have not been fully investigated. We have extended a standard olfactory receptor model to mixtures and found through its mathematical analysis that odorant-evoked activity patterns are more stable across concentrations and first-spike latencies of receptor neurons are shorter for mixtures than for pure odorants. Shorter first-spike latencies arise from the nonlinear dependence of binding rate on odorant concentration, commonly described by the Hill coefficient, while the more stable activity patterns result from the competition between different ligands for receptor sites. These results are consistent with observations from numerical simulations and physiological recordings in the olfactory system of insects. Our results suggest that mixtures allow faster and more reliable olfactory coding, which could be one of the reasons why animals often use mixtures in chemical signaling.

## Author summary

Odorants are chemicals that bind to olfactory receptors, where they are transduced into electric signals. Although most natural olfactory stimuli are mixtures of several odorants, odor transduction has mainly been studied for pure odorants, and current models of odor transduction are inconsistent for mixtures. Here, we built a mathematical model of odor transduction that works consistently for both pure odorants and mixtures. Our analysis of the model revealed that for mixtures, responses are more stable across concentrations and are faster. Our findings suggest that due to the nature of odor transduction, mixtures are more effective stimuli than single odorants.

## Introduction

Most studies on olfactory processing have been performed with pure odorants [1–6] or with mixtures of few odorant components [7–12]. However, in natural environments, animals are typically confronted with odor cues that are mixtures containing numerous different odorants [13–16], and the signals used in chemical communication between animals are also predominantly mixtures [17,18]. While experiments with single odorants have provided valuable insights into the response profiles of receptors and olfactory processing in the brain, relying on single odorants alone to understand olfactory processing and coding may be problematic. For instance, it has frequently been advocated that odor identity is encoded combinatorially by the distributed response pattern across olfactory receptor types [1,19–22]. However, for single odorants, response patterns often change significantly when the concentration varies [2,23] which poses a challenge to concentration-invariant recognition of odor identity. Could the lack of concentration invariance for single odorants be ameliorated by more complex natural odors that are mixtures of many odorants?

In this work, we investigated whether and how responses to mixtures of multiple odorants may differ from responses to single odorants. We first extended a kinetic model of receptor binding and activation [7,24] to also consider mixture stimuli, resolving the known inconsistencies [9,25] in previous models that attempted a similar extension [7,26]. The simplicity and generality of our extended model allowed us to analyze the receptor dynamics for mixtures and single odorants in a broad, biologically realistic regime not limited to any particular animal species. We found that the steady state receptor activation patterns at low and high concentrations are more correlated for mixtures than for single odorants, which makes mixture responses more stable across concentrations in olfactory receptor neurons (ORNs) and AL output neurons (projection neurons, PNs). Furthermore, when the stimulus concentration is small, the fraction of activated olfactory receptors immediately after stimulus onset are higher for mixtures than for single odorants. These results hold both when the mixtures contain the same number of molecules as the single odorant, i.e. a mixture of A and B contains 50% A and 50% B and is compared against 100% A and 100% B, and when the components of the mixture have the same concentration as the single odorant, i.e. a mixture of A and B contains 100% A and 100% B. This larger receptor activation for mixtures leads to a shorter first-spike latency of ORNs in the low concentration regime. The reduced first-spike latency for mixtures is caused by the non-linear dependence of odor-receptor binding on odorant concentration described by the Hill coefficient, while the more stable response patterns across concentrations arise from the competition of the different odorants in a mixture for free receptor sites.

We next tested these results by numerical simulations of a simple computational model of the first stage of olfactory processing in insect brains, the antennal lobe (AL). The parameters in the model were tuned to match the statistics of olfactory responses in ORNs and PNs of honey bees to single odorants based on experimental data sets [1,27–29]. We verified that our analytical results for ORNs can also be observed in numerical simulations of the AL model. We then performed two pilot physiological experiments in *Drosophila* and honey bees and observed that the collected data are consistent with our models’ predictions. Finally, we observed that these novel insights also hold in the original model of Rospars et al [7] and hence are general consequences of two-stage receptor models and not specific to our proposed more consistent model. Overall, our results suggest that olfactory encoding for mixtures can be more rapid and more concentration invariant than for single odorants. Thus, the challenges of strong concentration fluctuations in natural odor plumes may be alleviated by the prevalence of mixtures and the nature of receptor dynamics.

## Results

### Receptor model for single-components and mixture stimuli

We first formulated a model for odor transduction by olfactory receptors that works consistently for both single odorants and their mixtures. The initial step of odor transduction involves the binding of odorants to receptors and the subsequent opening of ion channels in ORN dendrites. Accordingly, receptor dynamics has often been modelled by a 2-step binding and activation process [7,26], as shown in Eq 1.

**Fig 1:**
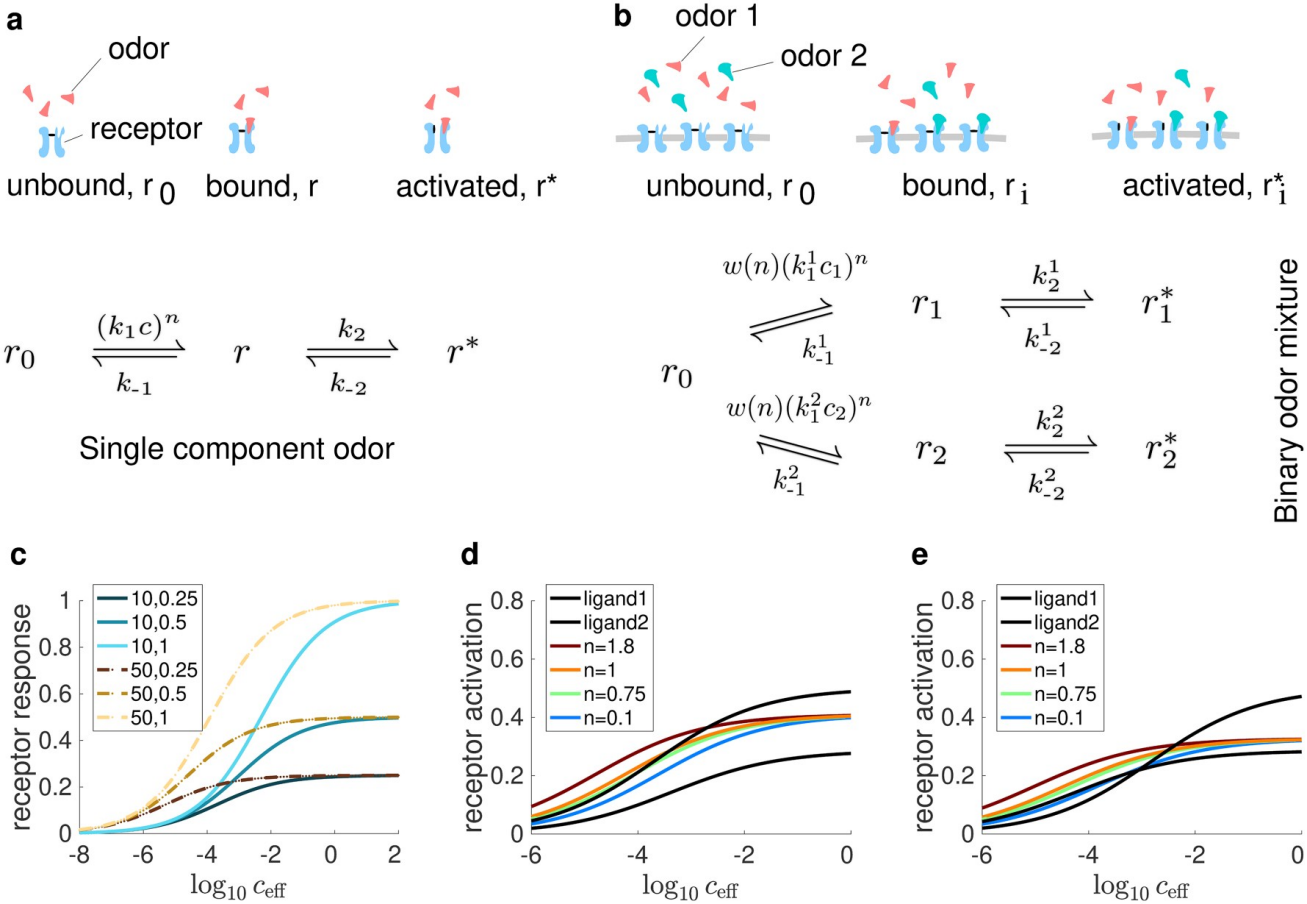
Analysis of the steady state receptor activation in our model for single odorants and mixtures at different concentrations. (a-b) Illustration for our model of binding of odor molecules to olfactory receptors and the activation process of the receptors for (a) single odorants (b) binary mixtures. (c) The receptor activation, r*, as described by Eq 3. In the limit of small *c*_eff_, receptors with the same *K*_eff_ are activated to the same degree, regardless of the value of $K_{2}^{\prime }$. In the limit of large *c*_eff_, receptor activation always approaches an asymptotic value depending on $K_{2}^{\prime }$, regardless of the value of *K*_eff_. Legend format: 1^st^ number: *K*_eff_, 2^nd^ number: $K_{2}^{\prime }$. (d-e) Examples of the response to binary mixtures as *n* varies. The activation in response to their constituent components are shown in black lines. In the limit of small *c*_eff_, receptor activation for mixtures can be synergistic, hypoadditive or suppressive depending on the values of *n*. In the other limit, the fraction of activated receptors for mixtures is independent of the value of *n* and always in between the fraction of activated receptors in response to the constituent components. In (d), component 1: $K_{2}^{\prime }=0.29$, *K*_eff_ = 8.33; component 2: $K_{2}^{\prime }=0.5$, *K*_eff_ = 20. In (e), component 1: $K_{2}^{\prime }=0.5$, *K*_eff_ = 8.33; component 2: $K_{2}^{\prime }=0.29$, *K*_eff_ = 20.

{r0˙=k-1r-(k1c)nr0r˙=(k1c)nr0-k-1r+k-2r*-k2rr˙*=k2r-k-2r*, (1)

where *k*_1_ (*k*_−1_) and *k*_2_ (*k*_−2_) are the (un)binding constants and (de)activation constants respectively; *c* is the concentration of the odor; and *n* is commonly known as the Hill coefficient (See below for more discussion). *r*_0_ and *r* refer to the fraction of unbound and bound (but not activated) receptors. The fraction of activated receptors, r*, “receptor activation” for brevity, determines the strength of excitatory input to ORNs. Before the onset of odorants, all receptors are in the unbound state (see ref [7]), i.e. we do not include spontaneous receptor activation [30]. This is illustrated in Fig 1a.

The rate of binding (*k*_1_*c*)^*n*^ is not linear with respect to the stimulus concentration if the Hill coefficient *n* is unequal to one, reflecting the experimentally observed non-linearity of the odor transduction process [7,28]. To consider receptor activation for mixtures, this model needed to be extended to multiple components. A simple implementation proposed by previous work [7,26] is to consider competition between odor molecules for receptor sites, and apply the transduction cascade to each of the components. This approach has two potential problems. First, the model predictions for receptor activation are inconsistent when we interpret a pure odor as a ‘mixture’ of identical components with arbitrarily partitioned concentrations and compare the results to the single component model in Eq 1 [9]. Second, the model cannot reproduce the different types of mixture responses observed experimentally [7,31] (This will be further discussed in the next sections). To deal with these issues, we propose that the overall binding rate depends on the linear sum of the components while the ratio of the binding rates of individual components remains the same as in the original model, as shown in Eq 2.

{r0˙=∑jk-1jrj-(∑jk1jcj)nr0r˙i=w(n)(k1ici)nr0-k-1iri+k-2iri*-k2irir˙i*=k2iri-k-2iri*, (2)

where $w(n)={\left(\sum_{j}k_{1}^{j}c_{j}\right)}^{n}/\sum_{j}{\left(k_{1}^{j}c_{j}\right)}^{n}$ and the subscript *i* indicates that the corresponding quantities describe the *i*^th^ component in the mixture. An illustrative description is shown in Fig 1b.

### Computational model of the honey bee antennal lobe

**Fig 2:**
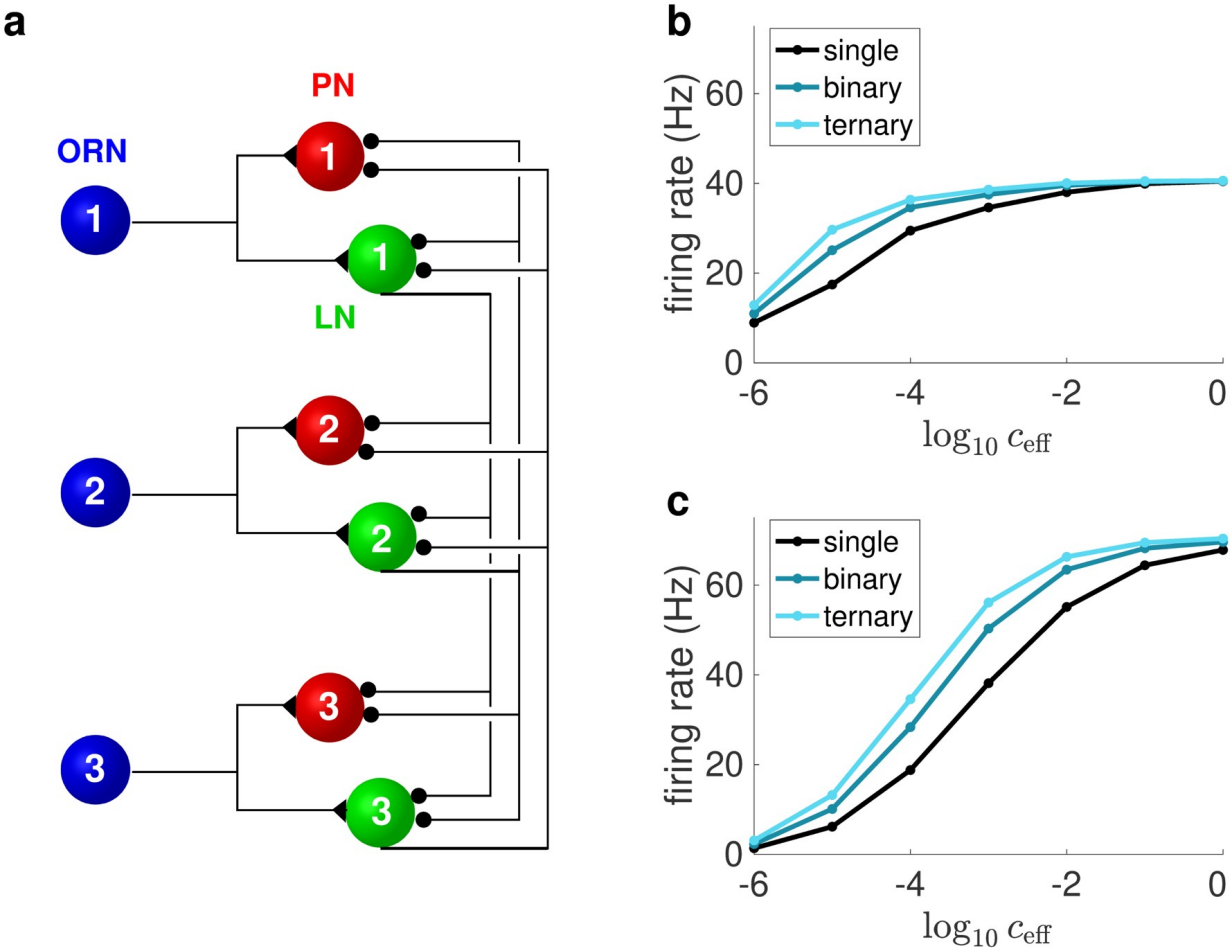
Average firing rate responses of ORNs and PNs to single odorants and mixtures. (**a**) Illustration of the model AL network. ORNs excite LNs and PNs of ‘their’ glomerulus, and LNs then project with inhibitory synapses to the PNs and LNs of other glomeruli. There are 160 ORN types and corresponding postsynaptic PNs and LNs in the full model, of which three are illustrated here. (**b-c**) The relationships between the stimulus concentration and the average firing rate response for (**b**) ORN and (**c**) PN, across all different odor-receptor combinations. The average responses for binary and ternary mixtures are larger than those for the components but smaller than twice and triple those of single odorants at low stimulus concentrations. They, however, become almost identical at high stimulus concentrations.

To assess the processing of olfactory stimuli beyond the receptor level we next built a model for the first olfactory area in the insect brain, the AL. Our model consists of 160 glomeruli, roughly equivalent to the experimentally observed number [32,33]. ORNs of the same type express only a single receptor type and respond with the same response profile. They project to the same glomerulus in the AL, where they synapse onto LNs and PNs. The response of an ORN depends on the receptor activation r* of the receptor type expressed by the ORN. LNs are local to the antennal lobe and modify the PN response pattern through lateral inhibition (Fig 2a). PNs project to higher brain centers such as the mushroom bodies and the lateral protocerebrum. In the model, responses from the same type of ORNs, LNs or PNs are approximated by their ensemble average, and are represented by a single unit. The firing rate of all units are approximated from a conductance-based leaky integrate-and-fire model with spike-rate adaptation [34].

The model reproduces key features of ORN and PN responses observed in separate experimental work that was not considered when building the model. It replicates the pulse tracking ability of ORNs [29] and the wide range of dose-response relationships in PNs [27]. Furthermore, this model supports the hypothesis that the observed decorrelation of glomeruluar PN response patterns to different odorants [27] and the statistical differences between ORN and PN responses [8] are predominantly caused by LN inhibition. Please refer to the S1 Appendix for details.

Using the receptor and AL models we described, we then analyzed whether and how the responses to single odorants and mixtures differ, as described in the next sections.

### Steady state receptor activation for mixtures and the role of the non-linear transduction process

In order to understand whether and how the receptor dynamics described by Eqs 1 and 2 may lead to qualitative differences between ORN responses to single odorants and mixtures, we first compared the model predictions for single odorants and mixtures at the receptor level. Unless otherwise mentioned, we are considering mixtures with equal absolute concentration *c* for all components. The steady state solution for receptor activation, r* and $r_{mix}^{*}$, in Eqs 1 and 2 can be expressed as

$$r^{*}=\frac{1}{\frac{1}{K_{2}^{\prime }}+\frac{1}{K_{\text{eff}}}\frac{1}{c_{\text{eff}}}}$$ (3a)

$$r_{mix}^{*}=\frac{1}{\frac{1}{K_{2}^{\text{mix}\prime }}+\frac{1}{K_{eff}^{mix}}\;\frac{1}{c_{eff}}},$$ (3b)

where $K_{1}=\frac{k_{1}^{n}}{k_{-1}}$, $K_{2}=\frac{k_{2}}{k_{-2}}$, $K_{2}^{\prime }=\frac{1}{\left(1+\frac{1}{K_{2}}\right)}$, *K*_eff_ = *K*_1_*K*_2_, $K_{eff}^{mix}=\frac{{\left(\sum_{j}k_{1}^{j}\right)}^{n}}{\sum_{j}{\left(k_{1}^{j}\right)}^{n}}\sum_{i}K_{eff}^{i}$, $K_{2}^{\text{mix}\prime }=\frac{1}{\sum_{i}\frac{p_{i}}{K_{2}^{i\prime }}}$, $p_{i}=\frac{k_{eff}^{i}}{\sum_{j}k_{eff}^{j}}$, $r_{\text{mix}}^{*}=\sum_{i}r_{i}^{*}$, *c*_*eff*_ = *c*^*n*^, and $K_{2}^{i\prime }$ and $K_{eff}^{i}$ refer to the value of $K_{2}^{\prime }$ and *K*_eff_ for the *i*^th^ odor component in the mixture stimulus. Please refer to S2 Appendix for the derivation of Eq 3.

We have studied receptor activation in response to odorants in the limit of low and high concentrations. When *c* is large, $\frac{1}{K_{2}^{\prime }}$ and $\frac{1}{K_{2}^{\text{mix}\prime }}$ dominate the denominator of Eqs 3a and 3b. When *c* is small, the terms containing $\frac{1}{K_{eff}}$ and $\frac{1}{K_{eff}^{mix}}$ dominate. The fraction of activated receptors is, therefore, determined by *K*_eff_ (or $K_{eff}^{mix}$ for mixtures) for low concentrations and $K_{2}^{\prime }$ (or $K_{2}^{\text{mix}\prime }$) for high concentrations. This is illustrated in Fig 1c.

It has been observed previously that ORN responses to mixtures can be superlinear to the sum of their components’ responses (synergetic), sub-linear to the sum but stronger than their weakest component’s responses (hypoadditive/suppressive) and weaker than their weakest component’s responses (inhibitory). This grouping and naming of mixture response types is slightly different from that used in [7,31,35] but it greatly simplifies our subsequent discussion below. Our model can produce synergetic and hypoadditive/suppressive receptor activation for mixtures. In the regime of small *c*, the interaction between odorant molecules is dominated by cooperative and suppressive transduction mechanics. In our receptor model, this is reflected by the additional factor *w*(*n*). By considering Eq 3 and taking the limit of *c* → 0, we found that these transduction mechanics are responsible for both hypoadditive/suppressive and synergistic mixture interactions in receptors: synergy can be achieved when *n* > 1, hypoadditivity/suppression when 0 < *n* < 1. When *n* = 1, the responses are strictly additive (Please refer to S3 Appendix for the derivation). To illustrate the role of n, we show a comparison of receptor activation in response to mixtures and to their components for two different combinations of $K_{2}^{\prime }$ and *K*_eff_, and different values of *n*, in Fig 1d and 1e. Note that even though it is possible to obtain inhibitory mixture interaction when *n* ≤ −1 (See S3 Appendix), we do not consider cases of non-positive *n*, as in such cases, the activation remains finite (when *n* = 0) or blows up (when *n* < 0) as *c* → 0, which is highly unrealistic. As such, our receptor model cannot reproduce inhibitory mixture interactions but, in fact, inhibitory responses are actually also very rare in insects (See Discussion).

Analysis of experimental data [28] shows that for most receptor types the Hill coefficient takes values between 0 and 1. This resonates well with the observation that responses to mixtures are predominantly hypoadditive/suppressive in data from previous experimental works [7,9,31].

In the limit of large *c*, receptor activation for a mixture is the weighted harmonic mean of the maximum of the receptor activation for its constituent components when they are present alone, and the weight $p_{i}=\frac{k_{eff}^{i}}{\sum_{j}k_{eff}^{j}}$ of component *i* is proportional to its activation gain at low concentrations. This implies that in this limit, mixture interactions must be hypoadditive/suppressive regardless of the value of *n*, as illustrated in Fig 1d and 1e. This result is supported by previous work [31], which showed that at high stimulus concentration, ORN responses were hypoadditive/suppressive in more than 97% of observed cases. This suggests that, at high concentration, the interaction between odor molecules of different types is dominated by their competition for free receptor sites, which gives rise to the suppression of mixture responses [7].

### Steady state receptor activation at the population level

Having established the different types of interactions for mixtures on the level of individual receptors, we next analysed the steady state receptor activation across the population of all receptor types. In Eq 3, the reaction rate parameters correspond to specific odor-receptor combinations. If we considered the entire space of possible odorant inputs and the space of all possible olfactory receptors, we would have a large number of possible odor-receptor combinations. Each combination *i* is characterized by parameters, $x_{1}^{i},\ldots ,x_{n}^{i}$, which are sampled from parameter sets *X*_1_, …, *X*_*n*_, each having the same number of elements as the number of possible odorant-receptor combinations. If we consider a sufficiently large number of such combinations, we may approximate $x_{1}^{i},\;\ldots ,\;x_{n}^{i}$, as random variables with some appropriate probability distribution each. We will take this view for all parameters in Eq 3 in this and the following section, which allows us to study the statistical properties of the activation of receptors across the population analytically. Note that we are not applying the above treatments to the Hill coefficient *n*, which reflects mainly properties of receptors and is assumed to be odorant-independent as supported by experimental observations [7,9].

Using this formalism, we found that at low concentration, the average receptor activation, across all glomeruli and all considered odorants, to binary (and ternary) mixtures is larger than to the single odorants but less than twice (and three times) those of single odorants. As shown in S3 Appendix, this derives from *n* being smaller than 1 for most receptors (see above). We used our antennal lobe model to study the average ORN and PN firing rates for single odorants, binary mixtures and ternary mixtures, using parameter distributions constrained by experimental observations (see Materials and methods), as shown in Fig 2b and 2c. We found that the above results regarding hypoadditivity/suppression of receptor activation for mixtures can readily be extended to ORN and PN firing rates.

While our antennal lobe model predicts roughly equivalent average firing rates in response to single odorants and mixtures at high concentrations, we cannot conclude that it is a general property of the structure of the receptor model (Eq 2). Rather, it could be a consequence of the parameter choices that were directly guided by experimental data from the honey bees’ olfactory system.

### The correlation between response patterns at high and low concentration is larger for mixtures

**Fig 3:**
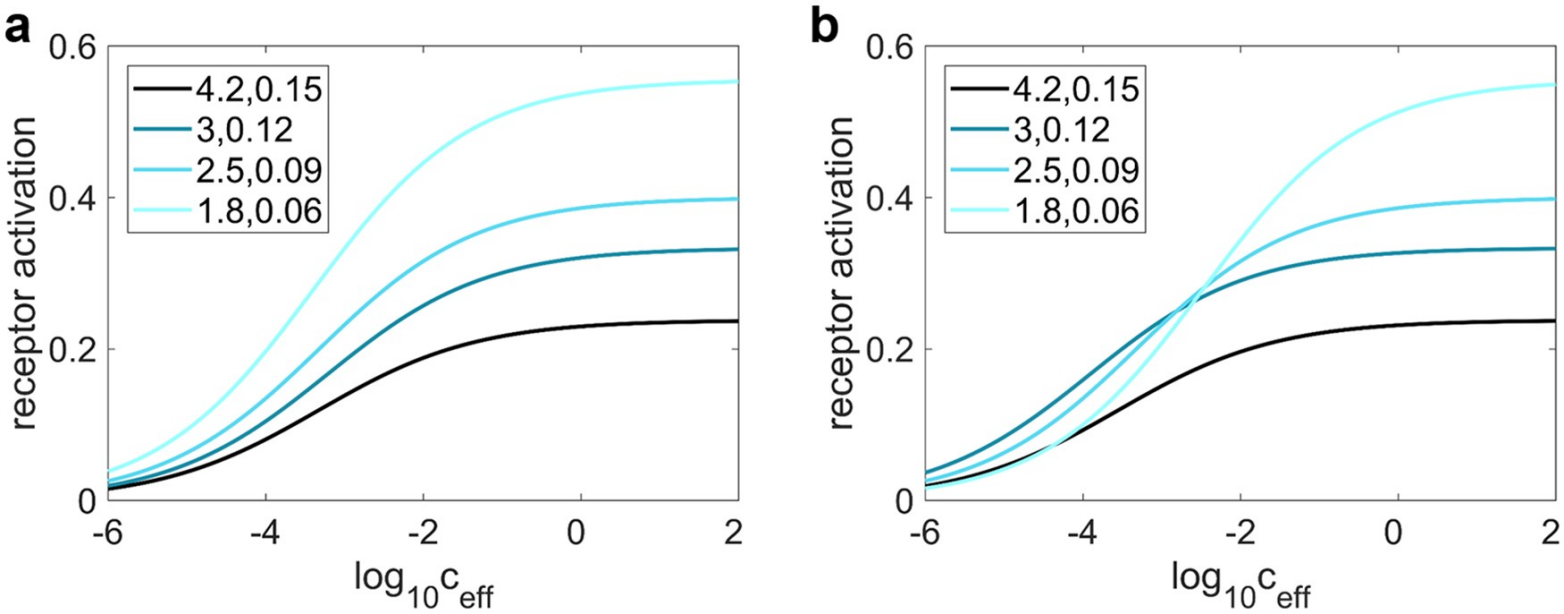
Receptor activation r* for receptors with different *K*_eff_ and $K_{2}^{\prime }$, where the correlation between the parameters are (a) strongly positive and (b) non-positive. When *K*_eff_ is strongly positively correlated with $K_{2}^{\prime }$, the fraction of activated receptors for different receptor types is roughly constant over a large range of effective concentrations, which indicates a high linear correlation between the activation patterns at different *c*_eff_. The opposite is observed if they are not positively correlated. Legend format: first number $\frac{1}{K_{2}^{\prime }}$, second number $\frac{1}{K_{eff}}$.

Based on our model, the fraction of activated receptors at the limit of low and high concentration is determined by *K*_eff_c_eff_ and $K_{2}^{\prime }$ ($K_{eff}^{mix}c_{eff}$ and $K_{2}^{\text{mix}\prime }$) for odors of a single (multiple) component(s), respectively, i.e. at low concentration, activation patterns essentially look like the pattern of *K*_eff_ values and at high concentration like the pattern of $K_{2}^{\prime }$ values. Accordingly, the correlation of the pattern of activation across receptor types at low and high concentration is essentially determined by the correlation of *K*_eff_ with $K_{2}^{\prime }$. If *K*_eff_ and $K_{2}^{\prime }$ are strongly positively correlated, weak activation for a given odor-receptor combination at low concentration is more likely accompanied by weak activation at high concentration, and vice versa (please refer to Fig 3 for an illustration).

At low stimulus concentration, the fraction of activated receptors for both single odorants and mixtures depends on the binding and activation rates of their components. At high concentration, however, it no longer depends on the binding rate in the case of single odorants, since essentially all available receptors are bound. Nevertheless, this is not the case for mixtures. As a result of odor molecules competing for receptor sites, the proportion of receptors bound to each component in a mixture depends on the competing ligands’ comparative binding affinities to the receptors. As the activation rate is not homogeneous for receptors bound to different ligands, the fraction of activated receptors hence depends indirectly on the binding rate of the components in mixtures, as in any two-stage competitive binding model. Here, this effect becomes evident by comparing the expression for $K_{2}^{\text{mix}\prime }$ and $K_{2}^{\prime }$ in Eq 3, where $K_{2}^{\text{mix}\prime }$ depends on the ‘effective binding rates’ of its components $K_{eff}^{i}$ while there is no dependence of $K_{2}^{\prime }$ on *K*_eff_.

We hypothesize that because of this indirect dependence of the fraction of activated receptors on binding rates for mixtures at high concentration, $K_{eff}^{mix}$ and $K_{2}^{\text{mix}\prime }$ would typically be more strongly positively correlated than *K*_eff_ and $K_{2}^{\prime }$. To test this hypothesis, we computed the correlations between $K_{eff}^{mix}$ and $K_{2}^{\text{mix}\prime }$, and *K*_eff_ and $K_{2}^{\prime }$ using a number of parameter sets with different ranges and statistical distributions, including biologically plausible ones, over many trials (Table 1). We were able to verify that our hypothesis holds true for all trials even if the distribution of *K*_1_ and *K*_2_ are skewed (Table 1). For constant Hill coefficient *n*, the correlation of $K_{eff}^{mix}$ and $K_{2}^{\text{mix}\prime }$ is always higher and there are only rare exceptions to this rule when *n* varies for different receptor types. (Table 1, rightmost column). Using Eq 3 and the conductance-based leaky integrate-and-fire model (see Materials and methods), we further showed in Table 1 that this higher correlation for $K_{eff}^{mix}$ and $K_{2}^{\text{mix}\prime }$ directly translates to higher correlations between ORN firing rate patterns for mixtures at low and high concentrations.

**Table 1:** 

Probability distribution	(min,max)/*μ*, *σ* for $k_{1}^{n}$	(min,max)/ *μ*, *σ* for *k*_−1_	(min,max)/ *μ*, *σ* for *K*_2_	Mean corr difference	% of discordant trials
$K_{eff}^{mix}$ **and** $K_{2}^{mix\prime }$, **and *K***_**eff**_ **and** $K_{2}^{\prime }$ **(*n* = 0.65)**					
Uniform	(0.5,5)	(0.005,0.05)	(0.01,1)	0.061	0
Exp(uniform)	(0.63,31.6)	(0.006,0.1)	(0.01,1)	0.095	0
Normal *	4,1.5	0.03,0.01	0.3,0.15	0.038	0
Uniform**	(0.5,5)	(0.005,0.05)	(1,10)	0.06	0
Uniform**	(0.01,0.1)	(0.1,1)	(0.01,1)	0.061	0
Exp(uniform)**	(0.01,1)	(0.01,1)	(0.01,10)	0.063	0
Log(uniform)**	(0.095,4.61)	(0.001,0.095)	(0.01,1.1)	0.042	0
**Average ORN firing rate for mixture and single odorant (*n* = 0.65)**					
Uniform	(0.5,5)	(0.005,0.05)	(0.01,1)	0.239	0
Exp(uniform)	(0.63,31.6)	(0.006,0.1)	(0.01,1)	0.379	0
Normal *	4,1.5	0.03,0.01	0.3,0.15	0.312	0
$K_{eff}^{mix}$ **and** $K_{2}^{mix\prime }$, **and *K***_***eff***_ **and** $K_{2}^{\prime }$ **(variable *n*)**					
Uniform	(0.5,5)	(0.005,0.05)	(0.01,1)	0.056	0
Exp(uniform)	(0.63,31.6)	(0.006,0.1)	(0.01,1)	0.096	0
Normal *	4,1.5	0.03,0.01	0.3,0.15	0.029	0
**Average ORN firing rate for mixture and single odorant (variable *n*)**					
Uniform	(0.5,5)	(0.005,0.05)	(0.01,1)	0.083	0.9
Exp(uniform)	(0.63,31.6)	(0.006,0.1)	(0.01,1)	0.308	0
Normal *	4,1.5	0.03,0.01	0.3,0.15	0.101	0.7

Numerical study of the difference in the mean correlation between *K*_eff_ and $K_{2}^{\prime }$ and that of $K_{eff}^{mix}$ and $K_{2}^{\text{mix}\prime }$, and the cross-concentration (*c* = 10^−4^ and *c* = 10^−1^) correlation between the response patterns, in terms of firing rate, to binary mixtures and single odorants over 1000 trials. The correlation of $K_{eff}^{mix}$ and $K_{2}^{\text{mix}\prime }$ is higher for all trials and for all choices of parameter sets. The variability of the “transduction constant” *n* (*n*′: log-normal distribution, *μ*_log*n*′_ = 0.44, *σ*_log*n*′_ = 0.22, *n*′ = *n*log10, chosen based on experimental measurements by Gremiaux et al (2012)), weakens the effects and introduces discordance in some of the trials. However, the cross-concentration correlation of the response patterns for mixtures is still significantly higher than that of single odorants and instances of discordance are rare. The consideration of independent distributions for $k_{1}^{n}$ and *k*_−1_ but not similarly for *k*_2_ and *k*_−2_ here is meant to introduce heterogenity to ‘stress-test’ our hypothesis. We have tried scenarios where we consider distribution of *K*_1_ as a whole, and also independent distribution for *k*_2_ and *k*_−2_. The results are qualitatively the same.

*A hard lower bound of 0 is imposed for unbounded distributions.

** Non-biologically plausible parameter sets

**Fig 4:**
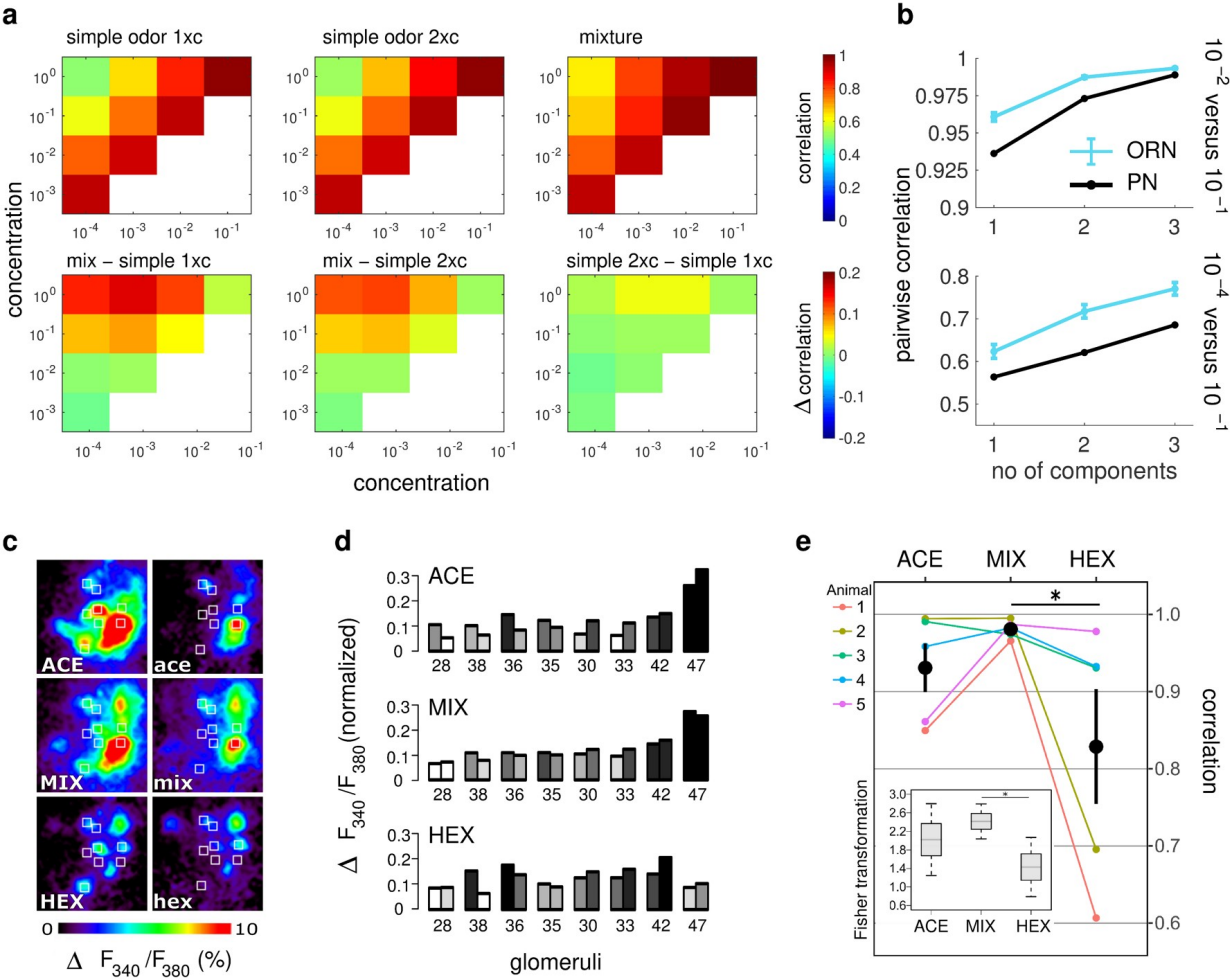
Pattern stability across concentrations for single odorants and mixtures. (**a**) Pearson’s correlation coefficients for ORN response patterns across various concentrations for single odorants and binary mixtures predicted by our AL model using constrained parameters as described in the Methods. They are calculated by averaging over all odorants in our model and over 1000 trials. Top panels from left to right: Correlation of a single odorant with itself, correlation of a single odorant with itself at twice the concentration, correlation of the mixture with itself; bottom row: differences in the corralations in the top row, between single odorant and mixture, between single odorants at twice the concentration and mixture, and between single odorant and single odorant at twice the concentration. The cross-concentration correlations for binary mixtures are higher. (**b**) Pearson’s correlation coefficients for ORN and PN response patterns at a low concentration (*c* = 3∙10^−*N*^ for single odorants; 1.5∙10^−*N*^ for binary mixtures;10^−*N*^ for ternary mixtures. Top: N = -2; Bottom: N = -4) and a high concentration (*c* = 3∙10^−1^ for single odorants; 1.5∙10^−1^ for binary mixtures; 10^−1^ for ternary mixtures) as predicted by our AL model. Concentrations were chosen so that the stimuli contained the same number of molecules each. Correlations were calculated as in **a** except that the results for PNs are from a single trial. The error bars for ORNs are the standard deviation across different trials. The correlation increases with the number of components in the stimuli for both ORN and PN. (**c**) Average AL response over 1.5s after odor onset of a representative animal as measured by calcium imaging. Left and right panels correspond to high and low concentrations respectively (one order difference). ACE: acetophenone; HEX: hexanol and MIX: mixture of ACE and HEX at 1:1 ratio. White squares mark the identified glomeruli used for the analysis. (**d**) Normalized activity elicited by odors at low and high concentrations (left and right columns in each pair). The gray scale used for the columns indicates the order of the glomeruli ranked according to the magnitude of their responses (lowest responding glomerulus: white; highest: black). The response pattern for mixtures is more stable than for both of its components, as illustrated by, in general, the higher similarity in height and color tone within each pair of bars (**e**) Pearson’s correlation coefficients between response patterns elicited by low and high odor concentrations. The correlation coefficients were calculated among patterns obtained by averaging three replicate measurements. Colored lines correspond to data of 5 bees. Black circles and error bars correspond to mean and SEM of the 5 bees. Statistical analysis (Wilcoxon one-sided signed rank test) was based on Fisher transformed correlation values (N_bee_ = 5; acetophenone-mixture, p = 0.16; 1-hexanol-mixture p = 0.03).

To determine whether this higher cross-concentration correlation between ORN response patterns for mixtures holds in more biological settings, we next studied the ORN firing rate response patterns predicted by our antennal lobe model, which uses statistically constrained parameter sets for the binding and activation constants, and Hill coefficients as observed experimentally in honey bees. We calculated the correlations between the steady state ORN response patterns across various concentrations of the same odorant, averaged over all odorants in our model and over 1000 trials. Fig 4a compares this correlation between single odorants and binary mixtures, and shows that the correlation for mixtures is higher, in particular when the difference in stimulus concentrations at which we compute the correlations is large. Note that this effect is not due to the higher number of molecules in mixtures than single odorants at the same concentration, as there are no notable changes to the above results even when we compensate this discrepancy in the number of molecules by doubling the concentration for single odorants. The cross-concentration correlation of PNs is also higher for mixtures than for single odorants, despite the presence of LN-mediated inhibition in the antennal lobe (Fig 4b). Finally, this correlation grows monotonically with the number of components in the mixture for both ORNs and PNs (For ORNs, we have also verified that the observed monotonic relationship between the cross-concentration correlation and the number of components holds for every trial).

To experimentally test the model prediction of higher cross-correlation for mixtures, we performed a pilot physiological experiment. We measured the PN response patterns to acetophenone and hexanol, and their mixture at high and low concentration in the honey bee antennal lobe, using calcium imaging (Fig 4c–4e). Calcium imaging was used because it allows simultaneous measurements of responses in several identifiable glomeruli. We observed that the cross-concentration correlation of responses to the mixture was indeed higher than for either of the mixture components for 4 out of 5 animals and on average the cross-concentration correlation of responses to the mixture was also higher (Wilcoxon one-sided signed-rank test, W = 3, p = 0.16 for acetophenone-mixture; W = 0, p = 0.03 for hexanol-mixture).

### The first-spike latency of ORNs is shorter for mixtures

Besides the overall firing rate pattern of glomeruli, the first spike latency of ORNs is of particular interest for fast odor detection (See eg [36]). The first-spike latency, defined as the time required for an ORN to fire the first spike after stimulus onset, is primarily determined by the initial receptor activation, r*(*t*) for small *t*, before the neuron fires the first spike. We consider the full sets of equations for single odorants and mixtures and find an approximation for r* and $r_{mix}^{*}$ at the limit of small *c* and *t*. In the approximation, we assume that $k_{1}^{n}\gg k_{-1}$ and $k_{1}^{n}\gg k_{2}$. This is realistic, because without these assumptions, the established high temporal resolution of ORN responses to repetitive odorant stimuli [29] (see also S1 Appendix) cannot be reproduced and the magnitude of ORN responses at different concentrations would be unrealistic. In the limit of short time after stimulus onset, we find (see S4 Appendix for the derivation)

$$r^{*}(t)≃k_{eff}c_{eff}\frac{t^{2}}{2}$$ (4a)

$$r_{mix}^{*}(t)≃k_{eff}^{mix}c_{eff}\frac{t^{2}}{2},$$ (4b)

where $k_{\text{eff}}=k_{1}^{n}k_{2}$ and $k_{eff}^{mix}=w(n)\sum_{i}k_{eff}^{i}$. To ensure that any differences in receptor activation are not caused by the disparity in the number of molecules present in the single odorants and in the mixture, we consider mixtures where each component has concentration *c*_0_, and single odorants at concentration *Nc*_0_, where *N* denotes the number of components. If the Hill coefficient *n* is smaller than 1, which holds for most receptors, we find that in the initial response (i.e. when *t* is small), the fraction of activated receptors for a mixture (Eq 4b) is larger than the average fraction of activated receptors for its constituent components with the same number of molecules (Eq 4a). This is a consequence of competing effects of non-linearity in mixture interaction, represented by the factor *w*(*n*), and odor transduction, represented by the non-linear scaling of receptor binding with stimulus concentration, *c*^*n*^. Under very general assumptions how latency depends on the fraction of activated receptors, this implies that the first-spike latency is shorter for the mixture (see S4 Appendix for the derivation).

**Fig 5:**
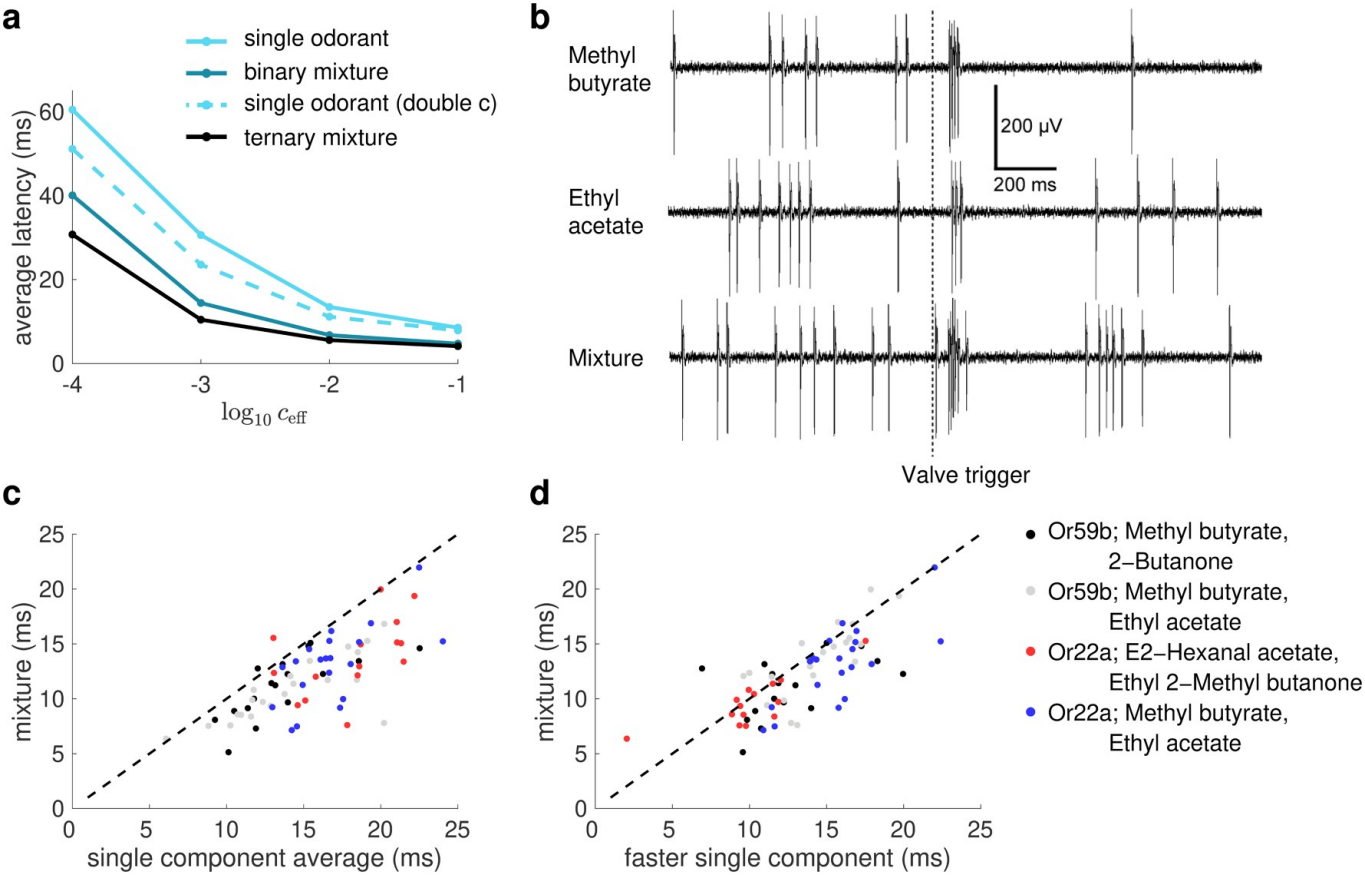
ORN first-spike latency for single odorants and mixtures. (**a**) The average first-spike latency predicted by our model ORNs decreases with the number of components in the odor stimulus. This effect is most pronounced when the stimulus concentration is low. The shorter first-spike latency for mixtures cannot be fully explained by the higher number of odor molecules in stimuli with more components compared to their counterparts with less components at the same concentration, since the latency for binary mixtures (dark cyan) is lower than that for a single odorant with doubled concentration (dashed line). Please note that all odor-receptor combinations with latency greater 100ms were taken to be 100ms when we calculate the average latency. (**b**) Example voltage traces obtained from recordings from an ab2 sensillum stimulated bymethyl butyrate, ethyl acetate and their mixture. The traces are filtered by a 100 to 3000 Hz bandpass. Large spikes belong to the OR59b-expressing ORN. (**c**) Recorded first spike latencies from *Drosophila* ORNs for binary mixtures and average latencies of their constituent components at doubled concentration, for 73 different animal-odor-receptor combinations. For the majority combinations (70 out of 73), the latency for mixtures is smaller than the average latency for the two components on their own. (**d**) Same as **(c)** but the comparison is made between mixtures and their constituent components with shorter latency at doubled concentration. The latency for mixtures is still smaller for most combinations (57 out of 73). The total number of measuremens for black/gray/red/blue is 17/20/21/15.

Fig 5a shows the average first-spike latency for all odorant-ORN combinations for single odorants, binary mixtures and ternary mixtures in our antennal lobe model. The average first-spike latency decreases with the number of components in the stimuli even after taking into account the discrepancy of the number of molecules as described in the previous paragraph.

To experimentally test the model prediction of shorter first-spike latencies for mixtures, we recorded spike responses of 2 different types of *Drosophila* ORNs to four binary mixtures and their constituent components using single sensillum recordings (Fig 5b) (We used *Drosophila* because recordings from identified ORNs are not currently possible in honey bees). In line with the results of our analysis in the previous paragraph and S4 Appendix, the first-spike latency to the mixture was shorter than the average of the first-spike latencies of their components at twice the concentration for most trials (70 out of 73, Fig 5c), which correspond to various animal-odor-receptor combinations. Fig 5d shows that for many of these combinations (57 out of 73), the first-spike latency for a mixture was even shorter than the latency for the constituent component which evoked the shorter latency.

### The enhanced robustness remains for mixtures with more components and also mixtures with components having unequal concentrations

Even though we have limited our analysis of cross-concentration correlations and responses latencies in this work to 2 and 3-component mixtures, one can easily verify, by repeating the analysis and simulation for other numbers of components, that cross-concentration correlations increase and response latencies decrease as the the number of components in a mixture increases. However, the results for more complex mixtures can already be predicted by considering the following argument: We can interpret a ternary mixture as a binary mixture of a binary mixture of two components with the third component. We can then consider the binary mixture as a single odorant by transforming $K_{2}^{\text{mix}\prime }$ and $K_{eff}^{mix}$ in Eq 3b into $K_{2^{\prime }}^{i\prime }$ and $K_{e\text{ff}}^{\prime }$, and apply the analyses in the previous sections with the third component being the second odor in the mixture (taking the value of $K_{2}^{2\prime }$ and $K_{eff}^{2}$). This procedure can be repeated to obtain results for mixtures having an arbitrary number of components. The mathematical proof for the validity of this approach is shown in the S2 Appendix (Eq. 29). Following this idea, one can clearly see that any of the considered changes in response properties with respect to the single odorant case must be monotonic as the number of components in the mixtures increases.

**Fig 6:**
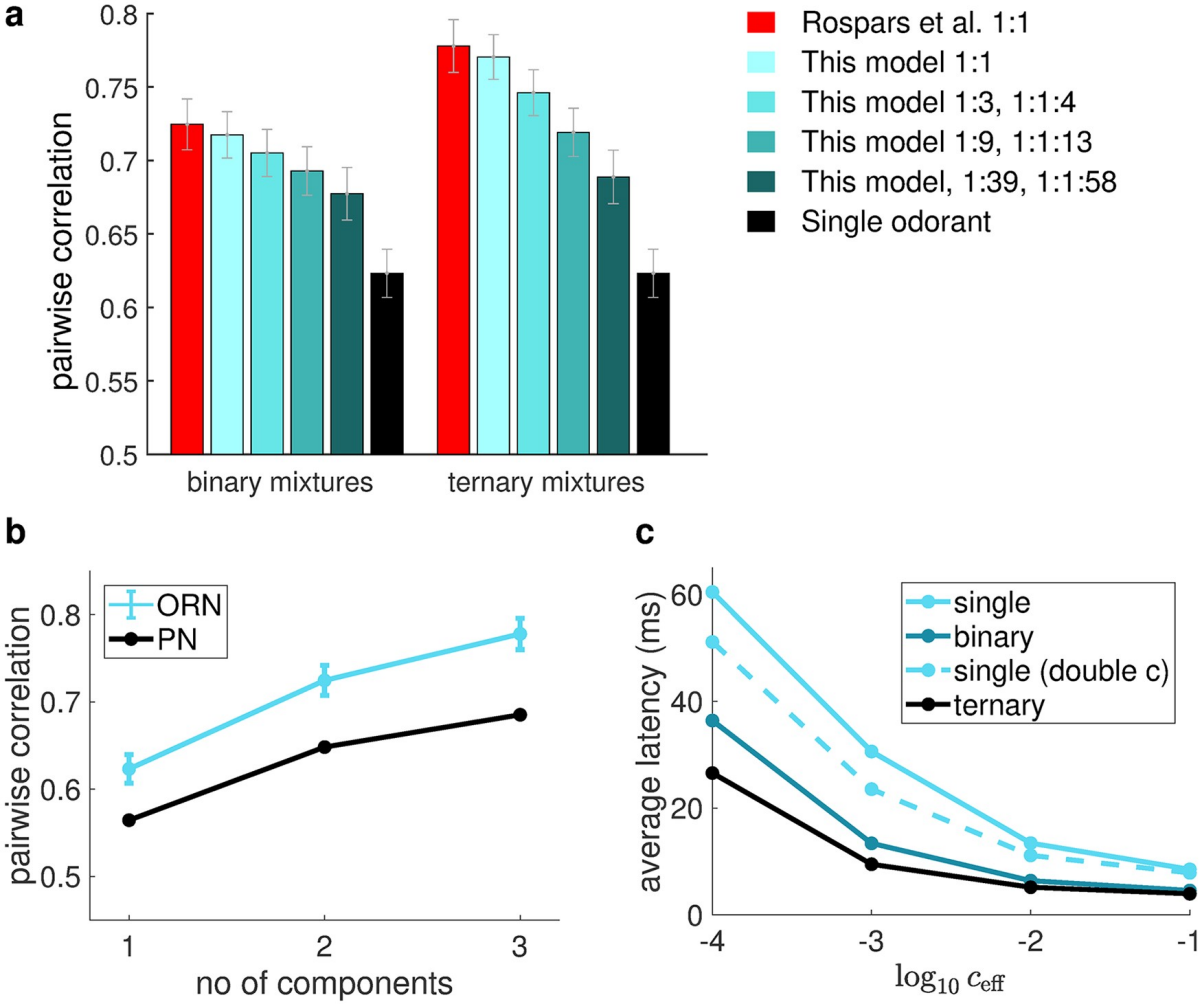
**(a)** Comparison between cross-concentration correlations of ORN response patterns for mixtures with their components under various ratios, computed using our receptor model. The size of the mixture effects will decrease smoothly as the ratio of components becomes more unbalanced, but the mixture effects can still be observed when the composition of the mixture is highly unbalanced (dark green). In addition, the mixture effects can still be observed even when considering previous models [7]. **(b)** Cross-concentration correlations of ORN response patterns and **(c)** first-spike latency for mixtures and single odorants when considering a previous model [7]. The mixture effects described in the main text can still be observed: Latency is shorter and cross-concentration correlations is higher as the no of components in the mixtures increase. Please refer to Fig 3b (bottom panel) and Fig 4a for a direct comparison with the results of our receptor models.

In the analysis of our model we only considered cases where the concentration of each component in the mixture is identical. If the concentrations of the components in a mixture are not identical, we can add weighting terms to the terms in the summation in $K_{eff}^{mix}$ and $K_{2}^{\text{mix}\prime }$, so that $K_{eff}^{mix}$ becomes a weighted sum of $K_{eff}^{i}$ while the weight *p*_*i*_ in $K_{2}^{\text{mix}\prime }$ is further weighted by the effective concentration for different components (S2 Appendix, Eq. 26). Thinking heuristically, the pure odorants and their mixtures form a continuum from having a single odorant through unbalanced mixtures with just a very small proportion of a second odorant to a mixture of equal proportions. Receptor activation as calculated from Eq 2 will reflect this continuum and so will our results: As the solution for Eq 2 has no singularities as *c*_*i*_ varies, they will not be affected abruptly, but the size of the mixture effects will decrease smoothly as the ratio of components becomes more unbalanced. Numerical simulations confirm this as illustrated in Fig 6, which also shows that mixture effects can still be observed when the components in the mixture differ significantly in concentration.

### Our findings are qualitatively unchanged when using the previous, inconsistent model

The shorter response latency and the more stable response patterns across concentrations were deduced from a modified version of a standard receptor model [7,24,26] that was made consistent for self-mixtures by adding the non-linear term *w*(*n*). However, it is important to note that the results are not *caused* by the addition of this term, as explained below. The more stable response patterns across concentrations for mixtures hinges on competition between different ligands for receptor sites at high stimulus concentrations. Thus, it would be observed in any 2-step binding and activation model. For first-spike latency, it is clear from the derivation of Eq. 35 and 45 in S3 and S4 Appendices that the non-linear term *w*(*n*) actually leads to less activated receptors during the initial response to mixtures, which is, however, offset by the non-linear scaling of receptor binding and hence eventual fraction of activated receptors with stimulus concentration. Without the term *w*(*n*), the first-spike latency for mixtures would be even shorter. In essence, both more stable response patterns and shorter first-spike latencies hold for general two-stage receptor models and therefore can also be observed in previous models [7,26]. These deductions are verified by Fig 6, which show the results obtained using the models in ref [7].

## Discussion

We have extended a model of receptor binding and activation to mixtures, which can generate a large range of experimentally observed mixture interaction types [7,31] and addresses a known inconsistency of previous models [7] (see next section). The mathematical analysis of our model predicts qualitative differences between receptor activation for single odorants and for odorant mixtures, which leads to the ORN response patterns being more stable across concentrations and ORN response latencies being shorter for mixtures than for single odorants. These observations were confirmed in subsequent numerical simulations of a model of the honey bee AL and are consistent with our pilot physiological experiments in honey bees and *Drosophila*. A stronger verification of our predictions in physiological experiments will require testing many more different odorant combinations and is beyond the scope of this work.

### Comparison of our receptor model for mixtures with previous models

Previous work on extending the receptor model [7] led to conceptual inconsistencies, where the receptor activation for a single odorant, when interpreted as a mixture with itself, was unequal to the receptor activation for the same concentration of that odorant, when interpreted as a single odorant. In addition, the experimentally observed hypoadditive/supressive, synergistic and inhibitory mixture interactions [7,31] cannot be reproduced with the previous models [7]. By adding the non-linear term *w*(*n*), we now can reproduce hypoadditive/suppressive and synergistic receptor activation with our receptor model, but not inhibitory ones (see Discussion below). The factor *w*(*n*) relates to how odorants interact with receptors during the binding process when other odorants are present. As the nature of the chemical reactions involved in odorant receptors remains largely unknown for insects, we refrained from speculating on the biophysical mechanism corresponding to *w*(*n*).

Alternative solutions to the inconsistency problem have been proposed in previous works [9,25]. In [9], the receptor equations are linear throughout (which would correspond to *w*(*n*) = 1 in our model), and a non-linearity, described by a Hill coefficient and other parameters, is added to the steady state solution of these equations afterwards. [25] only considers steady state solutions, using different Hill coefficients for the same receptor type when it is stimulated by different odorants and their mixtures. It is non-trivial to ascertain whether and how the expressions for the steady state in [9,25] can be related to the steady state solutions of a consistent system of dynamical equations. Therefore, the methodology described in refs [9,25] does not allow us to study receptor activation outside the steady state regime. The analysis of initial receptor activation presented here, for example, would be impossible with this approach. A more recent study [35] presented a model of the olfactory transduction cascade in mammalian olfactory receptor neurons and considered the effect of masking, leading to weaker ORN responses for mixtures than for their weakest component. However, in insects, this kind of inhibitory mixture interaction is extremely rare [37–41]. For example, in the Colorado potatoe beetle, inhibitory mixture interactions were observed in only 1 out of 117 odorant-receptor combinations [41]. The mechanism of masking in the model in [35] is based on alterations of cAMP binding affinity. However, in contrast to mammalian receptors, which are metabotropic, insect olfactory receptors act as ligand gated channels [42,43], with delayed, metabotropic auto-regulation [44] and the masking mechanism described in [35] does not apply. This difference might explain the lack of observations of masking and inhibitory mixture effects in insects.

### Strengths and limitations of our AL model

Our antennal lobe model is not built by directly fitting individual ORN responses to honey bee data, but by matching the statistics of responses (such as mean, standard deviation, and correlations of responses) across different receptor types and a wide range of odorants, between the model and experiments. This allows us to model the full receptor repertoire of honeybees in spite of limited data and study the statistical properties of their responses to different types of stimuli, with the trade-off that a generated model glomerulus may not correspond to any particular glomerulus in a honey bee. Generally speaking, experimental data of brain activity is often variable due to many factors, e.g. noise, experience-dependent plasticity and possible genetic diversity within a species, and such data is seldom complete. It may, therefore, often be more productive to reproduce the statistics of the observed responses, rather than detailed measurements of individual cells, especially if coding strategies are based on the overall activity pattern across many different types of sensory cells [2,8,20].

We used the responses of 28 glomeruli to 16 odors [1] to estimate the statistics of the fraction of activated receptors of honey bee receptors. Honey bees have 160 glomeruli, and corresponding receptor and ORN types. There are three studies, which measured responses to a similar or the same set of odorants as in [1] in additional 35 to 43 honey bee glomeruli [5,45]. These studies reported similar response properties in the other glomeruli as in the 28 glomeruli selected for this study. We, therefore, are confident that the wide range of response statistics observed in the 28 glomeruli of [1] is sufficiently large and typical to generalize to other glomeruli.

When generating the ORN responses, we assumed that all the interactions take place at the receptor level, i.e. an ORN would integrate input from receptors expressed on its dendrites, but ORNs do not interact with each other. Experimental measurements have shown that for a minority of odorant-receptor combinations, ORN responses can decrease with concentration [6,46]. A plausible explanation for this observation are non-synaptic interactions between ORNs. It has been reported in *Drosophila* that excitation of an ORN can inhibit its neighbor(s) in the sensillum, typically of other types, via ephaptic interactions [47,48]. This may cause the response of an ORN type to weaken as odorant concentration increases if its neighbors’ responses strongly increase with concentration. However, it is unclear whether the same effects would exist in other animals, which have different sensilla structure. Tailoring the model to a specific animal to include, e.g. ephaptic interactions, would require more detailed consideration of the sensilla structure of the said animal and is beyond the scope of this work.

### The role of the 2-step binding and activation process in olfactory receptors

There are two distinct chemical processes taking place in olfactory receptors: binding of odorant molecules to receptors and activation of bound receptors [7]. Our model results (Eq 3) elucidate how the olfactory response depends on each process. In the limit of low stimulus concentration, we may consider the combination of the binding and activation process to be a single effective binding process, with an ‘effective binding rate constant’ *K*_eff_ (or $K_{eff}^{mix}$ for mixtures). However, in the limit of high stimulus concentration substantial differences become apparent as essentially all receptors are bound. For single odorants, receptor responses depend mainly on the activation process, mathematically evident from $K_{2}^{\prime }$ being independent of the rate constant *K*_1_. For mixtures, however, $K_{2}^{\text{mix}\prime }$ does depend on the values of *K*_1_ of the mixture components. This implies, as we have shown, that the two-stage process reduces the correlation between the response patterns induced by a single odorant at different concentrations but preserves more of this correlation for mixtures. Having a two-stage process, hence, appears adaptive for recognizing mixture stimuli in the face of strong variations in overall concentration as observed in natural odor plumes. As shown and discussed in the section ‘Our findings are qualitatively unchanged when using the previous, inconsistent model’, these results are not a consequence of our modified mixture model but apply more generally to two-stage receptor binding models.

### Implications of our results for olfactory coding

An important question is how the lower average first-spike latency and higher correlation between responses across concentrations for mixtures affect coding of olfactory information, e.g. odor identity. Behavioral experiments [49,50] suggest that odor identification can be achieved on the time scale of a few 10s to 100 milliseconds. What coding schemes are possible under such temporal constraints? One possibility would be to sample response patterns for a fixed amount of time, after which a decision about odor identity is made [51–53]. With lower latency, more ORNs could be recruited for the identification of a particular odor. This implies larger information capacity of the system for mixture stimuli. Another possibility would be to determine the odor identity by the responses of a fixed number of ORNs [53,54]. In this case, the lower average latency for mixtures allows them to be identified by the system more quickly.

In natural environments, odorant molecules move through turbulent fluids (air or water) in filaments, forming complex odorant plumes, which results in rapid and unpredictable fluctuations in the concentration of odors encountered by animals [55,56]. Therefore, to identify an odor, the response of the olfactory system needs to be robust against changes in odor concentration. The higher correlation between response patterns across concentrations for mixtures than for single odorants is therefore conducive to odor identification.

One may argue that such correlations hinder the coding of odor concentration. There are several alternatives of how concentration information can be coded, as discussed in previous work. For instance, information for concentration may be coded by other features of the response, like the proportion of activated glomeruli [3,57], or by utilizing special connectivity patterns between different sensory units acquired through learning [58]. It is also possible that concentration information is encoded by the temporal patterns of input, for instance the first-spike latency of all or a subset of units [59] or the degree of synchrony between the firing of different units [60]. Therefore, the improved identity coding due to more invariant response patterns at steady state does not necessarily compromise concentration coding, but it remains an open question how exactly identity and concentration coding may be simultaneously achieved [3,59,61].

## Materials and methods

### Antennal lobe model of honey bees

Our model consists of 160 receptor types, roughly corresponding to the number observed in honey bees [32,33]. The receptor activation patterns in the model in response to odor stimuli were generated in a multi-stage process. The steady state activation of 28 olfactory receptor types for time-invariant odor inputs at saturating concentration to 16 different odors was directly adopted from corresponding experimental measurements of glomerular responses with bath-applied Ca^2+^ dyes at high odor concentrations [1]. We then generated the activation of the remaining 132 receptor types to the same 16 odors using a method inspired by previous work [62]. The activation patterns are generated from a combination of previously generated activation patterns, including the data in ref [1], and noise. The ratio of the combination is determined by a target similarity matrix of odor activation patterns and a global variable which determines the overall amount of correlations across the activation patterns. The parameters are chosen such that the statistical distribution of the pairwise correlations of receptors across odors in the generated activation patterns matches that of the 28 receptors adopted from data. The generated activation patterns are then rescaled such that the mean and the variance of the activation patterns for all receptor-odor combinations of the activation patterns across odors for each receptor match the experimentally observed values in [1].

Receptor responses to chemically similar odors are correlated [5]. In our model, such correlations are quantified using the normalized Euclidean distance *d*_ij_ between the response vectors of two different odors *i* and *j*, denoted by *x*_*i*_ and *x*_*j*_.

$$d_{ij}=\sqrt{\frac{\sum_{k}{\left(x_{ik}-x_{jk}\right)}^{2}}{N}},$$ (5)

where *N* is the total number of receptor types and the subscript *k* labels the different receptor types. The steady state activation of all previously generated receptor types are then iteratively tuned so that the Euclidean distance matrix *d* for the generated activation patterns matches the distance matrix observed in the experimental data. The tuning processes are designed to limit changes to the statistical quantities calibrated previously.

Upon completion of this process, we have determined the steady state activation of receptor types in response to stimuli at high, time-invariant concentration. The dynamical receptor activation for each odor-receptor combination to stimuli at arbitrary concentration are then generated by Eqs 1 and 2. To obtain the values of the parameters in these equations, we note that the steady state activation in response to a time-invariant stimulus with respect to stimulus concentration can be described by Hill curves [7] (see also S2 Appendix).

$$g(C)=\frac{g_{max}}{1\;+\;exp[n\;{log}_{10}(C-C_{\frac{1}{2}})]},$$ (6)

where *g* is the ORN response, and *C* = log_10_*c* is the logarithm (to base 10) of the concentration *c*. *g*_max_, the Hill coefficient *n* and the inflection point $C_{\frac{1}{2}}$ provide partial constraints on parameters in Eqs 1 and 2. *n* and $C_{\frac{1}{2}}$ are sampled from log-normal and normal distributions as experimentally observed [28], while *g*_max_ corresponds to the amplitude of the steady state activation generated previously. In dealing with the remaining degrees of freedom, we take into account the typical timescale of dynamics in the antennal responses measured experimentally [29].

### AL network

In our model, ORNs provide excitatory input to PNs and GABAergic local interneurons (LNs), and all ORNs of a given type project to the same glomerulus. PNs also receive inhibitory input from LNs of all other glomeruli (Fig 2a). To be consistent with previous findings [63], the connections from the LN in glomerulus *j* to the PN and LN in glomerulus *i* have a weight *w*_*ij*_, which is a function of the correlations *ρ*_*ij*_ between the corresponding ORN response patterns,

$$w_{ij}=(1-\delta_{ij})[w_{0}+H(\rho_{ij})\times \rho_{ij}w_{corr}],$$ (7)

where *δ*_*ij*_ is the Kronecker delta, *H* is the Heaviside step function, *w*_0_ and *w*_corr_ are normal distributed random variables, and *ρ*_*ij*_ is the Pearson correlation between the response of ORN *i* and *j* when stimulated by odors at high concentration, as obtained in the previous section:

$$\rho_{ij}=\frac{Cov({x^{T}}_{i},{x^{T}}_{j})}{\sigma ({x^{T}}_{i})\sigma ({x^{T}}_{j})}.$$ (8)

The strengths of ORN-LN and ORN-PN connections are uniform. The strengths of all connections are then jittered by a small amount of noise. Please refer to Table 1 for the details of the parameters.

### Conductance-based leaky integrate-and-fire model

To obtain the firing rate response of ORNs, LNs and PNs, we approximated the dynamics of a neuron by a conductance-based leaky integrate-and-fire model with adaptation [34,64].

$$\tau_{\text{eff}}(t)\frac{dV}{dt}=-V+RI_{\text{eff}}(t)-{RI}_{\text{adapt}}(t)$$

$$\tau_{\text{adapt}}\frac{dI_{\text{adapt}}(t)}{dt}=-I_{\text{adapt}}(t)$$

$$I_{adapt}=I_{adapt}^{max}\;\text{at}\;t=t_{f},$$ (9)

where *V* is the membrane potential, *R* is the membrane resistance, and *I*_adapt_ is the adaptation current, which is set to $I_{\text{adapt}}^{\text{max}}$ just after firing events at *t*_*f*_ and decays exponentially with decay time constant *τ*_adapt_. *I*_eff_ and *τ*_eff_ are the effective input current and effective membrane time constant having taken into account the conductance effects [64]. They are described by

$${RI}_{\text{eff}}(t)=\frac{{V_{e}(g}_{e}(t)+{V_{i}g}_{i}(t)+V_{r}g_{l}}{g_{\text{total}}(t)},$$

$$\tau_{\text{eff}}(t)=\tau_{m}/g_{\text{total}}(t),$$

$$g_{\text{total}}(t)=1+g_{e}(t)+g_{i}(t),$$ (10)

where *V*_*r*_ is the membrane rest potential, *V*_*e*_ and *V*_*i*_ are the reversal potentials of excitatory and inhibitory synapses, *g*_*l*_ is the membrane leak conductance, *g*_total_(*t*) is the total conductance of the neuron, and *g*_*e*_ and *g*_*i*_ are the excitatory and inhibtory conductances. For ORNs, $g_{e}=g_{\text{ORN}}\sum_{i}r_{i}^{*}$, where *g*_ORN_ is a constant, and *g*_*i*_ = 0. For PNs and LNs, *g*_*e*_ and *g*_*i*_, as a first order approximation, are proportional to the firing rate of ORNs and LNs [65]. For PNs, we also add constant background input into *g*_*e*_(*t*) and *g*_*i*_(*t*) for both ORNs and PNs so that they fire spontaneously at 5–20*Hz*. We then set *R* = 1 by absorbing it into other variables. When *V* reaches the threshold *V*_th_, the neuron fires a spike and *V* is immediately reset to *V*_reset_.

We then adopted the adiabatic approximation by considering the input to be quasi-stationary on the time scale of neuronal firing, such that *τ*_eff_(*t*) and *I*_eff_(*t*) are taken to be constants. With the additional assumption of noise-free input and setting *t*_*f*_ = 0, the membrane potential before the next firing event can be obtained analytically as follows:

$$V=V_{\text{reset}}e^{\frac{-t}{\tau_{\text{eff}}}}+I_{\text{eff}}(1-e^{\frac{-t}{\tau_{\text{eff}}}})-\frac{\tau_{\text{adapt}}I_{\text{adapt}}^{\text{max}}}{\tau_{\text{adapt}}-\tau_{\text{eff}}}(e^{\frac{-t}{\tau_{\text{adapt}}}}-e^{\frac{-t}{\tau_{\text{eff}}}}),$$ (11)

The instantaneous firing rate of the neuron can then be obtained using:

$$\nu =\frac{1}{t_{\text{thres}}+t_{\text{refract}}},$$ (12)

where *t*_thres_ is the time when *V* = *V*_th_, which is to be obtained numerically, and *t*_refract_ is the absolute refractory period. We then used Eqs 11 and 12 to calculate *v* at different time points for fluctuating input. Please note that *v*, by our definition, does not directly relate to temporal information of spike patterns, e.g. the inter-spike interval between any given pair of spikes.

In this work, we take $I_{\text{adapt}}^{\text{max}}=I_{\text{adapt}}^{\text{base}}\sqrt{\sum_{i}r_{i}^{*}}$ for ORNs, and $I_{\text{adapt}}^{\text{max}}=I_{\text{adapt}}^{\text{base}}\sqrt{\nu_{pre}}$ for PNs and LNs, where $I_{\text{adapt}}^{\text{base}}$ is a constant and *v*_pre_ is the firing rate of the corresponding units in the previous iteration. However, qualitatively similar results can be obtained by assuming $I_{\text{adapt}}^{\text{max}}$ to be constant.

All parameter values for the model can be found in Table 2.

**Table 2:** Parameters used in the AL model.

Random variables		p.d.f.*	value/*μ*, *σ*	units	remarks
**Parameters for the Hill curves in** (2) **and receptor dynamics**					
$C_{\frac{1}{2}}$		normal	-3, 1		hard boundary: $-4.4<C_{\frac{1}{2}}<-0.4$
*n*′		log-normal	0.45, 0.3		hard boundary: 0.7 < *n*′ < 3.5
*k*_1_		normal and then scaled	1.2, 0.15	ms^-1^	scaling factor: $\frac{1}{\sqrt{10^{x}\;}},\;x=\frac{{C_{\frac{1}{2}}\;n}^{'}}{log10}$ hard boundary: 0.1 < *k*_1_ < 5000
*k*_2_		normal	0.1, 0.01	ms^-1^	hard boundary: *k*_2_ > 0
Note: *k*_−1_ and *k*_−2_ are constrained by the above. Hard boundary: *k*_−1_ > 0.01, 0 < *k*_−2_ < 50					
**AL network connectivity between units** **(Note in all cases hard boundaries of mean ± 2 standard deviations are applied)**					
*w*_0_		normal	0.006, 0.002		
*w*_corr_		normal	0.01, 0.001		
*g*_ORN_		constant	2	nS	
ORN-PN		normal	0.045, 0.01		
ORN-LN		normal	0.013, 0.003		
LN-PN		normal	0.04, 0.01		
LN-LN		normal	0.004, 0.001		
**Spiking model**					
*τ*_*m*_		constant	20	ms	
*V*_*e*_		constant	50	mV	
*V*_*i*_		constant	-75	mV	
*V*_*r*_		constant	-70	mV	
*g*_*l*_		constant	1	nS	
*V*_*th*_		constant	-50	mV	
*V*_reset_		constant	-70	mV	
*t*_refract_		constant	2	ms	
background excitation	ORN	constant	0.28	nS	
	PN	normal	0.24, 0.02	nS	hard boundary: mean ± 2 sd
background inhibition	ORN	constant	0.5	nS	
	PN	normal	0.15, 0.01	nS	hard boundary: mean ± 2 sd
$I_{\text{adapt}}^{\text{base}}$	ORN	constant	40	mA	
	PN	normal	4.5, 0.4	mA	hard boundary: mean ± 2 sd
	LN	normal	1.8, 0.2	mA	hard boundary: mean ± 2 sd
*τ*_adapt_	ORN	constant	60	ms	
	PN/LN	constant	25	ms	

*probability density function

### First-spike latency of ORNs

The first-spike latency of neurons, defined by the time taken for the neuron to fire the first spike after stimulus onset, cannot be obtained from the instantaneous firing rate. Instead, we directly integrate Eq 9 numerically, assuming that *V* takes a mean value *V*_mean_, which is based on background inputs, at *t* = 0 and obtain the first-spike latency by finding the time *t* at which *V* = *V*_th_. For the purpose of this calculation we were still assuming noise-free input. We also assumed that the neurons have not been stimulated before, and since we are only considering the period until the first spike occurs, we used *I*_adapt_ = 0. An absolute latency of 1ms is added to the latency generated by our simulation to mimic the time required for the diffusion of odor molecules in the sensilla.

### Single sensillum recordings in *Drosophila*

Experiments were performed on female 1–9 days old *Drosophila melanogaster* wild type Canton S flies. The flies were raised at 25 °C on a standard *Drosophila* medium, with a 12/12 h day/night cycle. Single sensillum recordings were performed on large basiconic ab2 and ab3 sensilla of the left antenna. The flies were fixed in plastic pipette tips, and the left antenna was glued with low melting wax (1:1:1 mixture of n-Eicosan, myristic acid and dental wax) to get access to the medial side. The recording and reference electrodes were tungsten wires (diameter = 0.1mm), which were electrolytically sharpened with AC-current in a 0.5 M KOH solution. The recording electrode was inserted into the sensillum with a micromanipulator (Kleindieck). The reference electrode was inserted into the complex eye. Signals of the recording electrodes were differentially amplified against the reference electrode using 1000x gain and bandpass-filtered between 1 and 8,000 Hz (MA 103 and MA 102, Universität zu Köln). Noise from the powerline was reduced by a Hum Bug (Quest Scientific), and signals were digitized by a Micro 3 1401 (CED) A/D converter. Odorant stimuli were controlled using the Spike2 software (version 7.03; CED). The identity of sensilla was determined by their morphology, and also by comparing their responses to diagnostic odorants (methyl acetate, 2-butanone, isobutyl acetate and ethyl butyrate; all diluted 1:1000 in mineral oil) with previously reported responses [66].

Odorant stimuli were generated by opening the valves of a custom made olfactory stimulator [67] for 20 ms. The interstimulus interval was 60-70s. Pure odorants were stored in glass vials and the headspace was drawn into an air dilution system in which a defined amount of odorized air could be removed and replaced by clean air via flowmeters (042-15-GL for the first dilution step, 112-02GL for the 10x dilutions, Analyt-MTC). The rate of air flow per odorant channel was 300 ml/min and the total rate of air flow at the outlet of the stimulator was 2.1L/min resulting in an airspeed of 1.2 m/s. We used methyl butyrate, ethyl acetate, 2-butanone, E2-hexenyl acetate, Ethyl 2-methylbutanoate (all Sigma-Aldrich) as odorants at concentrations X and 2*X, with X being the minimum concentration at which spike rate responses could be measured reliably. The minimum concentration was adjusted for each odorant-receptor combination by drawing air from the headspace of pure odorants in vials with different cross-sectional areas (the larger the cross-sectional area, the more odorant molecules can evaporate into the headspace) and by diluting the odorant headspace in clean air (See Table 3). Throughout the experiment the odorant vial was constantly flushed with air so that the headspace concentration reached steady state.

**Table 3:** 

ORN	Odorant	Dilution (×10^−3^)	Cross-section area of vial (cm^2^)	Air flow through vial (ml/min)
OR59b	Methyl butyrate	4.17	3.1	250
	2-Butanone	3.67	3.1	220
OR59b	Methyl butyrate	2.50	3.1	150
	Ethyl acetate	0.15	0.8	9
OR22a	E2-hexenyl acetate	4.17	3.1	250
	Ethyl-2-methylbutanoate	3.67	3.1	220
OR22a	Methyl butyrate	2.07	3.1	124
	Ethyl acetate	2.00	15.9	120

Settings for creating minimum odorant concentrations X for the single sensillum recordings in *Drosophila* in Fig 4c.

Binary mixtures were generated by opening the two odorant channels simultaneously, such that the concentration of a given odorant was the same when it is the sole stimulus and when it is a part of a mixture stimulus. For each sensillum, we measured its responses to all three types of stimuli (mixture and both of its constituent components) at both concentrations once in a single recording session. A total of 73 different animal-odor-receptor combinations were recorded.

### Calcium imaging in honey bees

Honey bee, *Apis mellifera*, pollen foragers were obtained from regular hives located at the campus of the University of Buenos Aires, Argentina. The bees were briefly cooled on ice and restrained in individual holders. After recovery from cooling, the bees were fed with 1.0 M sucrose solution and left undisturbed until staining at the evening of the same day. A window was cut into the head capsule posteriorly to the joints of the antennae and anteriorly to the medial ocellus. PNs were stained by backfilling their axons with the calcium sensor dye Fura-dextran (potassium salt, 10,000 MW; Invitrogen, Eugene, USA). The tip of a glass microelectrode coated with dye was inserted into both sides of the protocerebrum, dorsolateral of the α-lobes where the lateral antenno-protocerebral tract enters the lateral calyces of the mushroom bodies [68,69]. The dye dissolved into the tissue in 3 to 5 seconds. The window was closed immediately using a piece of formerly removed cuticle and sealed with Eicosane (Sigma-Aldrich). After staining, the bees were fed and left undisturbed for 10 to 16 hours. After that, both antennae were fixed pointing towards the front using Eicosane. The head capsule was opened again and the brain was rinsed with saline solution to remove all extracellular dye (in mM: NaCl, 130; KCl, 6; MgCl_2_, 4; CaCl_2_, 5; sucrose, 160; glucose, 25; and HEPES, 10; pH6.7, 500 mOsmol; all chemicals from Sigma-Aldrich). Glands and trachea covering the ALs were removed. Only ALs that were stained homogeneously across all visually accessible glomeruli were used for imaging. Only one AL per bee was measured. Body movements that could affect imaging recordings were suppressed by gently compressing the abdomen and thorax with foam. In addition, a second hole in the head capsule was cut between the antennae and the mandibles, and the compact structure of muscles, esophagus and supporting chitin was lifted and put under slight tension. After preparation, the bees were mounted on the microscope and were allowed to recover for 20 minutes before imaging. Imaging experiments for all animals consisted of 12 odor stimulations separated by 1 minute each. Two measurements were made for each of the three types of stimuli (1-hexanol, acetophenone (both from TCI America, Portland OR) and their binary mixture) at two different concentrations in random order. Odors were delivered using a custom designed odor delivery device, which provided single odorants or mixtures at defined concentrations. The device had five independent odor channels that were activated briefly in pairs to create a stimulus. Each channel was connected to the headspace of a different bottle. The bottles contained 1-hexanol diluted 1:10(a) or 1:100(b) in mineral oil, acetophenone diluted 1:10(c) or 1:100(d) in mineral oil, and mineral oil only(e). Each bottle was connected to the respective odor channel by a solenoid valve which could be opened and closed synchronously with others using the imaging acquisition software TillVision (Till Photonics). In this work, the odors used were the combination of: a/e (for high concentration of 1-hexanol); b/e (low concentration 1-hexanol); c/e (high concentration acetophenone); d/e (low concentration acetophenone); a/c (high concentration mixture) and b/d (low concentration mixture). All odor channels converged into a mixing chamber, where the odors from the two opened channels were mixed. The mixed odors were then further diluted in a main air-stream, which also delivered them to the bee antennae. The main air-stream had a flow rate of 500 ml/min, while that of an odor channel is 50ml/min. Thus, the real concentration of an odor reaching the bees was actually $\frac{1}{10}$ of the concentration measured in the odor channel. An exhaust located 10 cm behind the bee removed the odors continuously.

Calcium imaging was performed using an EMCCD iXon camera (ANDOR, Belfast, UK) mounted on an upright fluorescence microscope (Olympus BX-50WI, Japan) equipped with a 20× dip objective, NA 0.95 (Olympus). Filter- and mirror-set: 505 DRLPXR dichroic mirror and 515 nm LP filter (Till-Photonics, Gräfelfing, Germany). Excitation light with alternating wavelength of 340 and 380 nm was generated by a Polychrome V (Till-Photonics). Acquisition protocols were made using the software TillVision (Till-Photonics). The sampling rate was 8 Hz. The spatial resolution was 125×125 pixels binned on a chip of 1000×1000 pixels. The intensity of the fluorescence lamp was controlled by the imaging acquisition software such that the exposure times to 340 and 380nm excitation light were 20 ms and 5 ms respectively. Images were analyzed using software written in IDL (Research Systems, CO, USA) by Giovanni Galizia (University Konstanz, Germany) and in R by Emiliano Marachlian [69]. Each measurement produced two sequences of 96 fluorescence images; one obtained for 340 nm excitation and another one for 380 nm excitation light ($F_{340}^{i}$ and $F_{380}^{i}$, where *i* is the image index, ranging from 1 to 96). For each of the 96 pairs of images, we calculated pixel-wise the ratio: *R*^*i*^ = *F*^*i*^_*340*_*/F*^*i*^_*380*_. Afterwards, we subtracted from all *R*^*i*^ the background ratio *R*_*b*_, defined by the average ratio *R*^*i*^ from 1s before odor onset to odor onset.

$$R^{i}=\frac{F_{340}^{i}}{F_{380}^{i}}-R_{b}\;for\;i\geq 24$$ (13)

$$R_{b}=\frac{\sum_{i=16}^{23}\frac{F_{340}^{i}}{F_{380}^{i}}}{8}$$ (14)

*R*^*i*^ represents the difference of fluorescence in window *i* to the fluorescence in the reference window and is proportional to a change in the intracellular calcium concentration. The analysis of odor induced activation patterns in the present study was based on signals from 8 glomeruli that were identified across all bees on the basis of their morphology and positions using the published atlas of the honey bee AL [32,33]. Glomeruli are visible in the raw fluorescence images at 380 nm excitation light after backfilling the PNs with FURA (Fig 4c). The level of glomerular activation was calculated by averaging the activity *R*^*i*^ in a square area of 7×7 pixels that correspond to 23×23 *μ*m and fits within the limits of each of the glomeruli. In this work, we did not consider the temporal structure of the response. Hence, the response is defined as the level of glomerular activation from 0 to 1.5 s after odor onset. Thus, odor-elicited activation patterns used for the analysis are 8-tuple vectors representing the average glomerular activity during the first 1500 ms after odor onset.

Responses from 5 different animals were recorded. Statistical analysis was performed using Wilcoxon one-sided signed-rank test.

## Supporting information

S1 Appendix Comparison of our AL model to other published experimental data. (DOCX)

S2 Appendix Derivation of the steady state receptor activation. (DOCX)

S3 Appendix Receptor activation in response to single component and mixture stimuli at low concentrations. (DOCX)

S4 Appendix Initial receptor activation and first-spike latency. (DOCX)

S1 Data Hill coefficients, binding and activation constants for the AL model. (XLSX)
