# Supplementary material for: Odorant mixtures elicit less variable and faster responses than pure odorants
Source: PLoS Comput Biol. 2018 Dec 10;14(12):e1006536. doi: 10.1371/journal.pcbi.1006536 (PMC6287832; doi:10.1371/journal.pcbi.1006536)
Supplement: S1 Appendix — (DOCX) [file pcbi.1006536.s001.docx]

**S1 Appendix: Comparison of our AL model to other published experimental data**

*A: Mean ORN response to pulsed input*

It has been shown that ORNs of honey bees can track pulses of odors up to high frequencies on the order of 100Hz [1]. When they are repeatedly stimulated by short odor pulses, their average responses, as measured by the electro-antennogram, exhibit oscillations with the same frequency as the odor pulses, defined as the reciprocal of the inter-pulse interval. The amplitude of the oscillations decreases with the pulse frequency (See Fig. A(a)).

We stimulated our model ORNs with short, $\text{sin}^{2}(t)$, odor pulses of ~5ms duration at different frequencies. Fig. A(a) shows that the average responses and their frequency-dependence are qualitatively similar to experimental measurements.

*B: Response-dose relationship of PNs at steady state*

While the ORN responses are almost always monotonically increasing with the concentration of the stimulus [2,3], it has been widely reported that this is not necessarily the case for PNs [2,4]. Experimental data [5] has illustrated that a wide range of individual response-dose relationships exists for PNs, which we can summarize as follows: The response monotonically increases/decreases with stimulus concentration (increase/decrease), the unit is inactivated for all concentrations (inactivated), or the response has a non-monotonic relationship with concentration (other). The fraction of occurrence of each type of relationship is shown in Fig. A(b).

Our model can reproduce all observed types of relationships. Note that the parameters of the model have been tuned to match the proportion with which each type of relationship occurred in the experimental data, but all the relationships can be reproduced within a large parameter space. The non-monotonic relationships arise from LN inhibition in the AL network. Without LN inhibition, only the ‘increase’ and ‘inactivated’ types of relationships can be recovered.

The presence of cases of responses being decreasing with stimulus concentration leads to a weaker scaling of the mean PN activity with concentration, in particular in the high concentration regime, where inhibition by LNs is strong. This has already been shown experimentally [6].

*C: Correlation between steady state PN responses*

It has been shown that while the steady state ORN responses across odorants are highly correlated [7], correlations of the corresponding PN response patterns are in general much weaker [5] (see also Fig. A(c)). Our model is designed to match the correlations of ORN responses observed in experimental data. It is of interest to study how well the correlations in generated PN responses match their experimental counterpart, which was not used in the construction of the model. Fig. A(d) shows the probability distribution of pairwise correlations between experimental and model ORN and PN responses across odorants. The model ORN responses are, as expected, highly correlated, but the degree of correlations is slightly less than that observed in experiments. The slight reduction in correlation likely arises as we tuned the model for chemical similarity (See Materials and Methods) and during spike generation. On the other hand, the distribution for PNs also exhibits a good qualitative match with its experimental counterpart, as both have a peak around correlation = 0. Please note that in Fig. A(b), the model responses are in the form of firing rates while the experimental responses are obtained from calcium imaging data, which is strongly related to the former but may not be directly proportional to it [8].

The decorrelation of the PN responses may occur in several distinct processes. Non-linearity in neuronal spiking models [9,10] and neural inhibition can both lead to decorrelation [11,12]. To elucidate the source of the decorrelation in our model, we compared the PN responses generated from AL networks with different inhibition paradigms. Fig. A(d) shows that the non-linearity in the LIF model, the inhibitory neural network involving LNs and the specific correlation-based PN-LN connectivity all contributed to the decorrelation in PN activities. However, the dominant effect is the presence of inhibition. This supports the hypothesis of previous experimental work [13]. The weak decorrelating effects due to non-linearity in neural spiking may be due to the introduced adaptation which makes the f-I curve of the LIF neuron more linear [14]. On the other hand, the effect of correlation based PN-LN connectivity is mainly reducing the amount of strongly correlated PNs by introducing strong mutual inhibition between them. Finally, we looked at the case when all the connections between the same unit type in the AL network are homogeneous (i.e. removing variability for all connection strengths). The results exhibit no major qualitative difference to the results where the LN-PN connections are uniformly distributed, suggesting that the heterogeneity in connectivity plays only a very minor, if not negligible, role in shaping the correlation across PNs.

*D: Comparison between ORN and PN responses*

Experimental measurements show that the correlation between receptor neuron and AL activity is around 0.6-0.7 [15]. We calculate the correlations between our model ORNs and PNs odorant by odorant, and they show a good match to experimental data. When LN inhibition is removed, the correlation increases but remains substantially smaller than unity. This suggests that both LN inhibition and the non-linearity of the LIF model contribute to the differences between ORN and PN response patterns.

We next investigate the phenomenon of ‘magnitude equalization’ in PN responses proposed by previous work [16]. In their work, the authors produce a model in which the mean of PN responses across glomeruli is less variable for different odorants than the mean of ORN responses. Here, we quantified this by the coefficient of variation (CV) of the mean firing rate across glomeruli for different odorants. Table A shows a drop in CV from the ORNs to the PNs, confirming the effect of PN ‘magnitude equalization’.

As in the last section, we compared the above results to the case where there is no inhibition. Under this circumstance, the ORN-PN correlation is significantly higher but still considerably smaller than unity (Fig. A(e)) while ‘magnitude equalization’ is weaker but clearly significant (Table A), suggesting that both the non-linearity in the LIF model and LN inhibition are important in producing both phenomena.

**Figure A: Comparison of our model results with experimental data on single odor stimulus**

| **a**  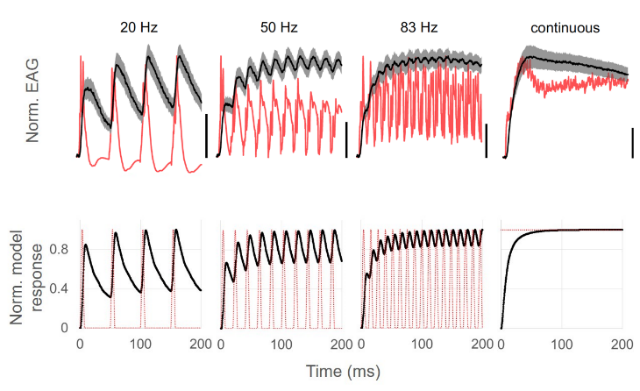 | **b**  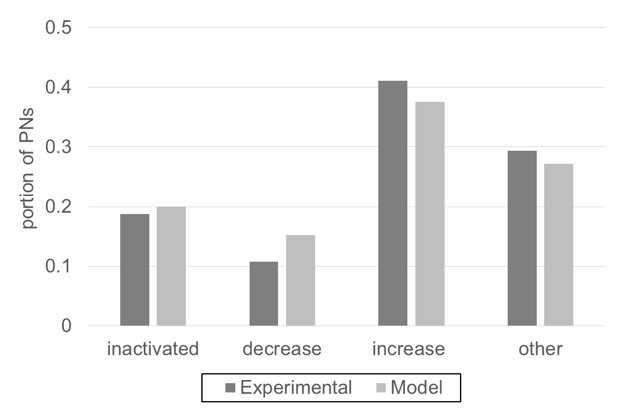 |
| --- | --- |
| **c**  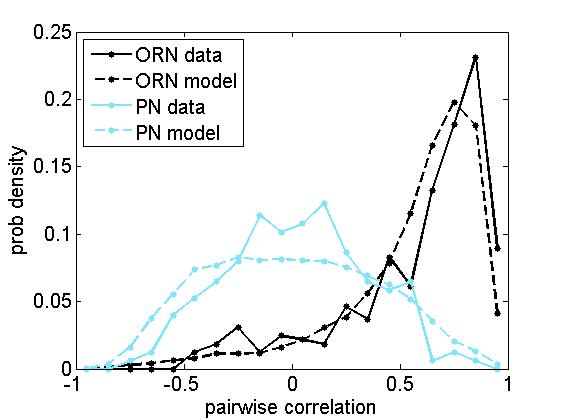 | **d**  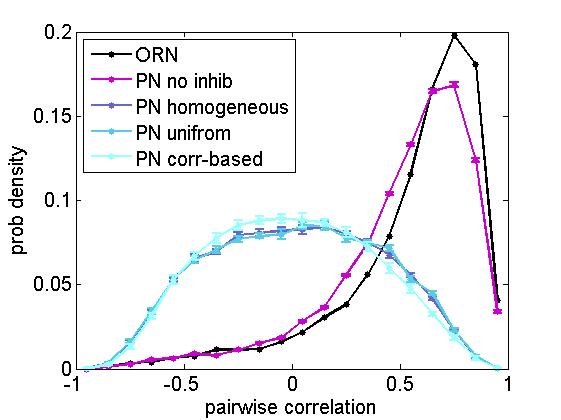 |
| **e**  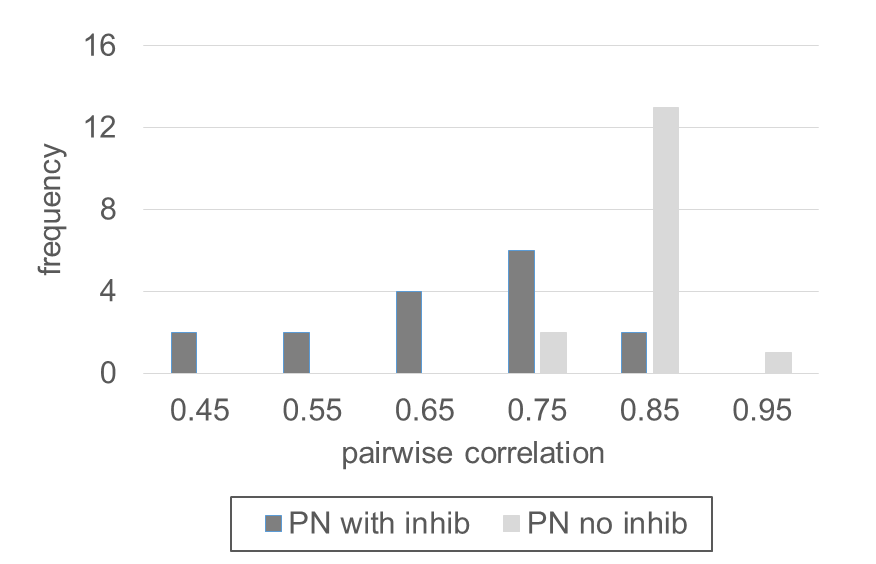 |  |

**Figure A**: Comparison of our model results with experimental data on single odor stimulus **(a)** ORN firing rate responses to pulsed and constant stimuli as measured by electro-antennogram recordings in Szyszka et al. (2014) (top) are qualitatively similar to the average normalized ORN responses to the corresponding stimuli (1-hexanol at $c={10}^{-4}$) predicted by our model (bottom). The red lines show the concentration of the input odor pulses. **(b)** Statistical distribution of different kinds of response-dose relationships observed in model PN responses (light grey bars) and experimental PN data from Ditzen (2005) (dark grey bars). We divide response-dose relationships into 4 different types: “inactivated” where PNs show no or very weak responses (<16Hz) to stimuli at any dose, “decrease” (“increase”) where responses decrease (increase) with dose and “other” where responses are independent of or display non-monotonic relationships with dose. All types of response-dose relationships can be generated by our model. With appropriate choice of parameters, the proportions of each type generated by our model can match those observed in experimental data. (**c**) Statistical distribution for pairwise correlations across response patterns for ORNs and PNs ($c=0.1$) observed in calcium imaging experiments [5,7] and our model. Our methods are designed to fit the distribution of correlation across ORNs observed in experimental data when building the model. No such fitting is done for the PNs. The ‘model responses’ correspond to the firing rate of model neurons. (**d**) Comparison between pairwise correlation across firing rate patterns for ORN and PN with no inhibition (purple), inhibition with homogeneous (blue), uniform and normal distributed (green) and correlation-based (light green) connectivity. The parameters for the latter three scenarios are chosen such that the mean output firing rate of PNs are the same for all the 3 cases. The results for PNs are obtained over 10 trials from the same set of ORN data and the error bars correspond to standard deviation across trials. $c=0.1$ for all the scenarios. **(e)** Statistical distribution of the pairwise correlation between the overall ORN and PN response for different odorant stimuli. The average correlation is around 0.6-0.7, which matches well with experimental observations. Note that if we remove the inhibitory effects of LNs, the correlation becomes higher but is still significantly below unity, which suggests that both the non-linearity in the LIF model and LN inhibition contribute to differences between ORN and PN response patterns.

**Table A: Mean firing rate of stimulated neurons in our AL model**

|  | **mean** | **std** | **CV** |
| --- | --- | --- | --- |
| ORN | 67.41 | 20.11 | 0.298 |
| PN with inhibition | 40.19±1.04 | 2.42±0.15 | 0.059±0.004 |
| PN no inhibition | 290.95±1.67 | 46.84±0.26 | 0.161±0.001 |

**Table A:** The mean firing rate for ORNs and PNs in the full model and for PNs without inhibition. All results for PNs are averaged over 10 trials. Entries format: mean ± standard deviation across the 10 trials.

**Reference**

1. Szyszka P, Gerkin RC, Galizia CG, Smith BH. High-speed odor transduction and pulse tracking by insect olfactory receptor neurons. Proc Natl Acad Sci U S A. 2014;111: 16925–30. doi:10.1073/pnas.1412051111

2. Sachse S, Galizia CG. The coding of odour-intensity in the honeybee antennal lobe: Local computation optimizes odour representation. Eur J Neurosci. 2003;18: 2119–2132. doi:10.1046/j.1460-9568.2003.02931.x

3. Carcaud J, Hill T, Giurfa M, Sandoz J-C. Differential coding by two olfactory subsystems in the honeybee brain. J Neurophysiol. 2012;108: 1106–1121. doi:10.1152/jn.01034.2011

4. Yamagata N, Schmuker M, Szyszka P, Mizunami M, Menzel R. Differential odor processing in two olfactory pathways in the honeybee. Front Syst Neurosci. 2009;3: 1–13. doi:10.3389/neuro.06.016.2009

5. Ditzen M. Odor concentration and identity coding in the antennal lobe of the honeybee Apis mellifera [Internet]. Freie Universität Berlin, Berlin. 2005. doi:10.1111/j.1752-1726.2008.00313.x

6. Krofczik S, Menzel R, Martin P. Rapid odor processing in the honeybee antennal lobe network. Front Comput Neurosci. 2009;2: 1–13. doi:10.3389/neuro.10.009.2008

7. Galizia CG, Sachse S, Rappert a, Menzel R. The glomerular code for odor representation is species specific in the honeybee Apis mellifera. Nat Neurosci. 1999;2: 473–478. doi:10.1038/8144

8. Grienberger C, Konnerth A. Imaging Calcium in Neurons. Neuron. Elsevier Inc.; 2012;73: 862–885. doi:10.1016/j.neuron.2012.02.011

9. de la Rocha J, Doiron B, Shea-Brown E, Josić K, Reyes A. Correlation between neural spike trains increases with firing rate. Nature. 2007;448: 802–806. doi:10.1038/nature06028

10. Rosenbaum R, Josić K. Mechanisms that modulate the transfer of spiking correlations. Neural Comput. 2011;23: 1261–1305. doi:10.1162/NECO_a_00116

11. Middleton JW, Omar C, Doiron B, Simons DJ. Neural Correlation Is Stimulus Modulated by Feedforward Inhibitory Circuitry. J Neurosci. 2012;32: 506–518. doi:10.1523/JNEUROSCI.3474-11.2012

12. Tetzlaff T, Helias M, Einevoll GT, Diesmann M. Decorrelation of neural-network activity by inhibitory feedback. PLoS Comput Biol. 2012;8: e1002596. doi:10.1371/journal.pcbi.1002596

13. Olsen SR, Wilson RI. Lateral presynaptic inhibition mediates gain control in an olfactory circuit. Nature. 2008;452: 956–960. doi:10.1038/nature06864

14. Ermentrout B. Linearization of F-I curves by adaptation. Neural Comput. 1998;10: 1721–1729. doi:10.1162/089976698300017106

15. Deisig N, Giurfa M, Sandoz JC. Antennal lobe processing increases separability of odor mixture representations in the honeybee. J Neurophysiol. 2010;103: 2185–2194. doi:10.1152/jn.00342.2009

16. Luo SX, Axel R, Abbott LF. Generating sparse and selective third-order responses in the olfactory system of the fly. Proc Natl Acad Sci. 2010;107: 10713–10718. doi:10.1073/pnas.1005635107
