# Supplementary material for: Odorant mixtures elicit less variable and faster responses than pure odorants
Source: PLoS Comput Biol. 2018 Dec 10;14(12):e1006536. doi: 10.1371/journal.pcbi.1006536 (PMC6287832; doi:10.1371/journal.pcbi.1006536)
Supplement: S2 Appendix — (DOCX) [file pcbi.1006536.s002.docx]

**S2 Appendix: Derivation of the steady state receptor activation**

Here we are deriving the steady state solution for Eqs. 1 and 2. The system of ordinary differential equations for binding and activation for a single odorant is given Eq. 1, which we show here again for convenience

$\left\{ \begin{aligned} \dot{r_{0}}=k_{-1}r-\left( k_{1}c \right)^{n}r_{0} \\ \dot{r} =\left( k_{1}c \right)^{n}r_{0} -k_{-1}r+k_{-2}r^{*}-k_{2}r \\ \dot{r^{*}}=k_{2}r-k_{-2}r^{*} \end{aligned} \right.$, (15)

At steady state, we have $\dot{r}_{0}=\dot{r}=\dot{r^{*}}=0$. Let $r_{0}+r+r^{*}=1$, this gives

$\left\{ \begin{aligned} r =K_{1}r_{0}c^{n} \\ r^{*}=K_{2}r \\ r_{0} =1-r-r^{*} \end{aligned} \right.$, (16)

where $K_{1}=\frac{{k_{1}}^{n}}{k_{-1}}, K_{2}=\frac{k_{2}}{k_{-2}}$.

Hence, we have

$r^{*}=K_{2}r=K_{1}K_{2}r_{0}c^{n}=K_{1}K_{2}\left[ 1-\left( 1+\frac{1}{K_{2}} \right)r^{*} \right]c^{n}$

$\left[ 1+K_{1}K_{2}\left( 1+\frac{1}{K_{2}} \right)c^{n} \right]r^{*}=K_{1}K_{2}c^{n}$

$r^{*}=\frac{K_{1}K_{2}c^{n}}{1+K_{1}K_{2}\left( 1+\frac{1}{K_{2}} \right)c^{n}}=\frac{1}{\frac{1}{K_{1}K_{2}c^{n}}+\left( 1+\frac{1}{K_{2}} \right)}$ . (17)

Let $K_{\mathrm{eff}}=K_{1}K_{2}$, ${K_{2}}^{'}=\frac{1}{\left( 1+\frac{1}{K_{2}} \right)}$, $c_{\mathrm{eff}}=c^{n}$, so that Eq. 17 can be rewritten as

$r^{*}=\frac{1}{\frac{1}{{K_{2}}^{'}}+\frac{1}{K_{\mathrm{eff}}}\frac{1}{c_{\mathrm{eff}}}}$ . (18)

We now consider, more generally, the system of equations for stimuli with an arbitrary number of components (Eq. 2):

$\left\{ \begin{aligned} \dot{r_{0}}=\sum_{j} k_{-1}^{j}r_{j}-\left( \sum_{j} k_{1}^{j}c_{j} \right)^{n}r_{0} \\ \dot{r}_{i} =\left( \sum_{j} k_{1}^{j}c_{j} \right)^{n}\frac{\left( k_{1}^{i}c_{i} \right)^{n}}{\sum_{j} \left( k_{1}^{j}c_{j} \right)^{n}}r_{0} -k_{-1}^{i}r_{i}+k_{-2}^{i}r_{i}^{*}-k_{2}^{i}r_{i} \\ \dot{r}_{i}^{*}=k_{2}^{i}r_{i}-k_{-2}^{i}r_{i}^{*} \end{aligned} \right.,$ (19)

where the subscript $i$ refers to the parameters for the $i^{\mathrm{th}}$ component in the stimulus.

At steady state, we have $\dot{r_{0}}=\dot{r}_{i}=\dot{r}_{i}^{*}=0 \forall i$. Let $r_{0}+\sum_{j} (r_{j}+r_{j}^{*})=1$, we have

$\left\{ \begin{aligned} r_{i} =w(n)K_{1}^{i}r_{0}c_{\mathrm{eff}}^{i} \\ r_{i}^{*}=K_{2}^{i}r_{i} \\ r_{0} =1-\sum_{j} \left( r_{j}+r_{j}^{*} \right) \end{aligned} \right.$, (20)

where $w\left( n \right)=\frac{\left( \sum_{j} k_{1}^{j}c_{j} \right)^{n}}{\sum_{j} \left( k_{1}^{j}c_{j} \right)^{n}}$. This gives

$r_{0}=1-\sum_{j} (r_{j}+r_{j}^{*})$

$\frac{r_{i}^{*}}{w\left( n \right)K_{1}^{i}K_{2}^{i}c_{\mathrm{eff}}^{i}}=1-\sum_{j} \left[ r_{j}^{*}\left( 1+\frac{1}{K_{2}^{j}} \right) \right]$

$\frac{r_{i}^{*}}{w\left( n \right)K_{\mathrm{eff}}^{i}c_{\mathrm{eff}}^{i}}=1-\sum_{j} \left( \frac{1}{{K_{2}^{j}}^{'}}r_{j}^{*} \right)$

$r_{i}^{*}+{K_{\mathrm{eff}}^{i}}^{'}c_{\mathrm{eff}}^{i}\sum_{j} \left( \frac{1}{{K_{2}^{j}}^{'}}r_{j}^{*} \right)={K_{\mathrm{eff}}^{i}}^{'}c_{\mathrm{eff}}^{i}$, (21)

where ${K_{\mathrm{eff}}^{i}}^{'}=w(n)K_{\mathrm{eff}}^{i}$ .

(21) can be rewritten in matrix form,

$M\boldsymbol{r}^{\boldsymbol{*}}=\boldsymbol{a}$, (22)

where $M_{ij}=\delta_{ij}+\frac{{K_{\mathrm{eff}}^{i}}^{'}c_{\mathrm{eff}}^{i}}{{K_{2}^{j}}^{'}}$, $\delta_{ij}$ is the Kronecker delta function, and $a_{i}={K_{\mathrm{eff}}^{i}}^{'}c_{\mathrm{eff},i} .$

$\boldsymbol{r}^{\boldsymbol{*}}$ can be obtained by finding the inverse of $M$, and the total fraction of activated receptors $r_{\mathrm{mix}}^{*}$ is given by $r_{\mathrm{mix}}^{*}=\sum_{i} r_{i}^{*}$.

First, we will look at the special case of binary mixtures. In this case,

$M=\left( \begin{matrix} 1+\frac{{K_{\mathrm{eff}}^{1}}^{'}c_{\mathrm{eff}}^{1}}{{K_{2}^{1}}^{'}} & \frac{{K_{\mathrm{eff}}^{1}}^{'}c_{\mathrm{eff}}^{1}}{{K_{2}^{2}}^{'}} \\ \frac{{K_{\mathrm{eff}}^{2}}^{'}c_{\mathrm{eff}}^{2}}{{K_{2}^{1}}^{'}} & 1+\frac{{K_{\mathrm{eff}}^{2}}^{'}c_{\mathrm{eff}}^{2}}{{K_{2}^{2}}^{'}} \end{matrix} \right)$ and

$M^{-1}=\frac{1}{\Delta}\left( \begin{matrix} 1+\frac{{K_{\mathrm{eff}}^{2}}^{'}c_{\mathrm{eff}}^{2}}{{K_{2}^{2}}^{'}} & \frac{-{K_{\mathrm{eff}}^{1}}^{'}c_{\mathrm{eff}}^{1}}{{K_{2}^{2}}^{'}} \\ \frac{{{-K}_{\mathrm{eff}}^{2}}^{'}c_{\mathrm{eff}}^{2}}{{K_{2}^{1}}^{'}} & 1+\frac{{K_{\mathrm{eff}}^{1}}^{'}c_{\mathrm{eff}}^{1}}{{K_{2}^{1}}^{'}} \end{matrix} \right)$, (23)

where the determinant $\Delta=\left( 1+\frac{{K_{\mathrm{eff}}^{1}}^{'}c_{\mathrm{eff}}^{1}}{{K_{2}^{1}}^{'}} \right)\left( 1+\frac{{K_{\mathrm{eff}}^{2}}^{'}c_{\mathrm{eff}}^{2}}{{K_{2}^{2}}^{'}} \right)-\left( \frac{{K_{\mathrm{eff}}^{1}}^{'}c_{\mathrm{eff}}^{1}}{{K_{2}^{2}}^{'}} \right)\left( \frac{{K_{\mathrm{eff}}^{2}}^{'}c_{\mathrm{eff}}^{2}}{{K_{2}^{1}}^{'}} \right)=1+\sum_{i} \frac{{K_{\mathrm{eff}}^{i}}^{'}c_{\mathrm{eff}}^{i}}{{K_{2}^{i}}^{'}}$. Therefore,

$\boldsymbol{r}^{\boldsymbol{*}}\boldsymbol{=}M^{-1}\boldsymbol{a}$

$=\frac{1}{\Delta}\left( \begin{matrix} 1+\frac{{K_{\mathrm{eff}}^{2}}^{'}c_{\mathrm{eff}}^{2}}{{K_{2}^{2}}^{'}} & \frac{-{K_{\mathrm{eff}}^{1}}^{'}c_{\mathrm{eff}}^{1}}{{K_{2}^{2}}^{'}} \\ \frac{{{-K}_{\mathrm{eff}}^{2}}^{'}c_{\mathrm{eff}}^{2}}{{K_{2}^{1}}^{'}} & 1+\frac{{K_{\mathrm{eff}}^{1}}^{'}c_{\mathrm{eff}}^{1}}{{K_{2}^{1}}^{'}} \end{matrix} \right)\left( \begin{matrix} {K_{\mathrm{eff}}^{1}}^{'}c_{\mathrm{eff}}^{1} \\ {K_{\mathrm{eff}}^{2}}^{'}c_{\mathrm{eff}}^{2} \end{matrix} \right)$

$=\frac{1}{1+\sum_{i} \frac{{K_{\mathrm{eff}}^{i}}^{'}c_{\mathrm{eff}}^{i}}{{K_{2}^{i}}^{'}}}\left( \begin{matrix} {K_{\mathrm{eff}}^{1}}^{'}c_{\mathrm{eff}}^{1} \\ {K_{\mathrm{eff}}^{2}}^{'}c_{\mathrm{eff}}^{2} \end{matrix} \right)$

$=\frac{1}{1+\sum_{i} \frac{{K_{\mathrm{eff}}^{i}}^{'}c_{\mathrm{eff}}^{i}}{{K_{2}^{i}}^{'}}}\boldsymbol{a}$ (24)

$r_{\mathrm{mix}}^{*}=\frac{\sum_{i} {K_{\mathrm{eff}}^{i}}^{'}c_{\mathrm{eff}}^{i}}{1+\sum_{i} \frac{{K_{\mathrm{eff}}^{i}}^{'}c_{\mathrm{eff}}^{i}}{{K_{2}^{i}}^{'}}}=\frac{1}{\frac{1}{K_{\mathrm{eff}}^{\mathrm{mix}}}\frac{1}{c_{\mathrm{eff}}^{1}}+\frac{1}{{K_{2}^{\mathrm{mix}}}^{'}}},$ (25)

where $K_{\mathrm{eff}}^{\mathrm{mix}}=\sum_{i} {K_{\mathrm{eff}}^{i}}^{'}\frac{c_{\mathrm{eff}}^{i}}{c_{\mathrm{eff}}^{1}}=w\left( n \right)\sum_{i} K_{\mathrm{eff}}^{i}\frac{c_{\mathrm{eff}}^{i}}{c_{\mathrm{eff}}^{1}}$, ${K_{2}^{\mathrm{mix}}}^{'}=\frac{1}{\sum_{i} \frac{p_{i}}{{K_{2i}}^{'}}}$,$p_{i}=\frac{K_{\mathrm{eff}}^{i}c_{\mathrm{eff}}^{i}}{\sum_{j} K_{\mathrm{eff}}^{j}c_{\mathrm{eff}}^{j}}$. (26)

If $c_{\mathrm{eff}}^{1}=c_{\mathrm{eff}}^{2}=c_{\mathrm{eff}}$, $K_{\mathrm{eff}}^{\mathrm{mix}}$ and $p_{i}$ can be further simplified as

$K_{\mathrm{eff}}^{\mathrm{mix}}=\sum_{i} {K_{\mathrm{eff}}^{i}}^{'}=w\left( n \right)\sum_{i} K_{\mathrm{eff}}^{i}$, (27)

and $p_{i}=\frac{K_{\mathrm{eff}}^{i}}{\sum_{j} K_{\mathrm{eff}}^{j}}$.

In fact Eq. 24 to 27 is true for mixtures with any number of components. Note that

$\left( M\boldsymbol{a} \right)_{i}=\sum_{j} \left[ \left( \delta_{ij}+\frac{{K_{\mathrm{eff}}^{i}}^{'}c_{\mathrm{eff}}^{i}}{{K_{2}^{j}}^{'}} \right){K_{\mathrm{eff}}^{j}}^{'}c_{\mathrm{eff}}^{j} \right]$

$={K_{\mathrm{eff}}^{i}}^{'}c_{\mathrm{eff},i}+\sum_{j} \left[ \left( \frac{{K_{\mathrm{eff}}^{i}}^{'}c_{\mathrm{eff}}^{i}}{{K_{2}^{j}}^{'}} \right){K_{\mathrm{eff}}^{j}}^{'}c_{\mathrm{eff}}^{j} \right]$

$=a_{i}\left[ 1+\sum_{j} \left( \frac{{K_{\mathrm{eff}}^{j}}^{'}c_{\mathrm{eff}}^{j}}{{K_{2}^{j}}^{'}} \right) \right]$ (28)

We have $M\boldsymbol{a}=\boldsymbol{a}\left[ 1+\sum_{j} \left( \frac{{K_{\mathrm{eff}}^{j}}^{'}c_{\mathrm{eff}}^{j}}{{K_{2}^{j}}^{'}} \right) \right]$, which gives

$\frac{1}{1+\sum_{j} \left( \frac{{K_{\mathrm{eff}}^{j}}^{'}c_{\mathrm{eff}}^{j}}{{K_{2}^{j}}^{'}} \right)}\boldsymbol{a}=M^{-1}\boldsymbol{a}=\boldsymbol{r}^{\boldsymbol{*}}$ (29)
