# Supplementary material for: Odorant mixtures elicit less variable and faster responses than pure odorants
Source: PLoS Comput Biol. 2018 Dec 10;14(12):e1006536. doi: 10.1371/journal.pcbi.1006536 (PMC6287832; doi:10.1371/journal.pcbi.1006536)
Supplement: S3 Appendix — (DOCX) [file pcbi.1006536.s003.docx]

**S3 Appendix: Receptor activation in response to single component and mixture stimuli at low concentrations**

When $c\to0$, $r^{*}$and ${r_{\mathrm{mix}}}^{*}$ in Eqs. 1 and 2 are given by

$r^{*}=K_{\mathrm{eff}} c_{\mathrm{eff}}$ (30)

${r_{\mathrm{mix}}}^{*}=K_{\mathrm{eff}}^{\mathrm{mix}}c_{\mathrm{eff}}=w\left( n \right)\sum_{i} K_{\mathrm{eff}}^{i}c_{\mathrm{eff}}$, (31)

Below, we will show that for mixtures with $N$ components,

$1\leq w\left( n \right)\leq N^{n-1}$ for $n\geq1$ and (32)

$1>w\left( n \right)>N^{n-1}$ for $n<1$, (33)

which would further imply that synergy can be achieved when $n>1$, hypoadditivity/suppression when $0<n<1$.

Since the parameters $k_{1}^{j}$ are strictly positive, we can rewrite $w\left( n \right)$ in terms of the $L^{p}$-norms of a vector $k_{1}=\left( k_{1}^{1}, \cdots, k_{1}^{N} \right)$,

$w\left( n \right)=\frac{\left( \sum_{j} k_{1}^{j} \right)^{n}}{\sum_{j} {k_{1}^{j}}^{n}}=\left( \frac{\sum_{j} k_{1}^{j}}{\left( \sum_{j} {k_{1}^{j}}^{n} \right)^{\frac{1}{n}}} \right)^{n}=\left( \frac{\left\| k_{1} \right\|_{1}}{\left\| k_{1} \right\|_{n}} \right)^{n}$, (34)

where $\left\| . \right\|_{p}$ corresponds to the $L^{p}$ norm.

It is straightforward to show, e.g. by Jensen’s inequality, that $\left\| . \right\|_{p}$ decreases with $p$, $\left\| k_{1} \right\|_{n}\leq\left\| k_{1} \right\|_{1}$ if and only if $n\geq1.$This gives

$\left\{ \begin{aligned} w\left( n \right)\geq1 \text{if } n\geq1 \\ w\left( n \right)<1 \text{otherwise} \end{aligned} \right.$ (35)

To obtain the upper (lower) bound for $n\geq1$ ($n<1$), we use Hölder’s inequality. For $n\geq1$, we have

$\sum_{j} \left( k_{1}^{j} \right)\left( 1 \right)\leq\left( \sum_{j} {k_{1}^{j}}^{n} \right)^{\frac{1}{n}}\left( \sum_{j} 1^{\frac{1}{1-\frac{1}{n}}} \right)^{1-\frac{1}{n}}$

$\sum_{j} k_{1}^{j}\leq N^{1-\frac{1}{n}}\left( \sum_{j} {k_{1}^{j}}^{n} \right)^{\frac{1}{n}}$

$\left( \sum_{j} k_{1}^{j} \right)^{n}\leq N^{n-1}\sum_{j} {k_{1}^{j}}^{n}$

$\frac{\left( \sum_{j} k_{1}^{j} \right)^{n}}{\sum_{j} {k_{1}^{j}}^{n}}\leq N^{n-1}$ (36)

For $n<1$, we have

$\sum_{j} \left( {k_{1}^{j}}^{n} \right)\left( 1 \right)\leq\left( \sum_{j} \left( {k_{1}^{j}}^{n} \right)^{\frac{1}{n}} \right)^{n}\left( \sum_{j} 1^{\frac{1}{1-n}} \right)^{1-n}$

$\sum_{j} {k_{1}^{j}}^{n}\leq N^{1-n}\left( \sum_{j} k_{1}^{j} \right)^{n}$

$\frac{\left( \sum_{j} k_{1}^{j} \right)^{n}}{\sum_{j} {k_{1}^{j}}^{n}}\geq N^{n-1}$ (37)

We stated that it is possible to obtain inhibitory interaction when $n<-1$. This can be illustrated with the example of $n=-2$ using the Cauchy-Schwarz inequality.

$\frac{\left( \sum_{j} k_{1}^{j} \right)^{-2}}{\sum_{j} {k_{1}^{j}}^{-2}}$ $=\frac{1}{\left( \sum_{j} k_{1}^{j} \right)^{2}\sum_{j} \left( \frac{1}{k_{1}^{j}} \right)^{2}}$ $\leq\frac{1}{\left( \sum_{j} \left( k_{1}^{j} \right)\left( \frac{1}{k_{1}^{j}} \right) \right)^{2}}$ $=\frac{1}{4}$ (38)

By Eq. 27 and 38, $K_{\mathrm{eff}}^{\mathrm{mix}}\leq\frac{1}{4}\left( K_{\mathrm{eff}}^{1}+K_{\mathrm{eff}}^{2} \right)$, which can be smaller than both $K_{\mathrm{eff}}^{1}$ and $K_{\mathrm{eff}}^{2}$.

On population level, if we assume that $w\left( n \right)$ and ${k_{1}^{i}}^{n}$ are essentially uncorrelated (while they are not truly independent, this is a good approximation since the former does not scale with $k_{1}^{i}$). Making use of the Eq. 36, we have

$N\left\langle r^{*} \right\rangle\geq\left\langle{r_{\mathrm{mix}}}^{*} \right\rangle\approx N\left\langle w\left( n \right) \right\rangle\left\langle r^{*} \right\rangle\geq N^{n}\left\langle r^{*} \right\rangle>\left\langle r^{*} \right\rangle,$ (39)

Similarly, it can be shown that $N\left\langle r^{*} \right\rangle\leq\left\langle{r_{\mathrm{mix}}}^{*} \right\rangle\leq N^{n}\left\langle r^{*} \right\rangle$if $n\geq1$.
