# Supplementary material for: Odorant mixtures elicit less variable and faster responses than pure odorants
Source: PLoS Comput Biol. 2018 Dec 10;14(12):e1006536. doi: 10.1371/journal.pcbi.1006536 (PMC6287832; doi:10.1371/journal.pcbi.1006536)
Supplement: S4 Appendix — (DOCX) [file pcbi.1006536.s004.docx]

**S4 Appendix: Initial receptor activation and first-spike latency**

The receptor dynamics equations for single odorant stimuli is given by Eq. 1, which we show here again for convenience.

$\left\{ \begin{aligned} \dot{r_{0}}=k_{-1}r-\left( k_{1}c \right)^{n}r_{0} \\ \dot{r} =\left( k_{1}c \right)^{n}r_{0} -k_{-1}r+k_{-2}r^{*}-k_{2}r \\ \dot{r^{*}}=k_{2}r-k_{-2}r^{*} \end{aligned} \right.,$ (40)

When $t\to0$ and with the assumption that ${k_{1}}^{n}>1\gg k_{-1} \mathrm{and} k_{2}$, $\dot{r}$ can be approximated by $\dot{r}\simeq{k_{1}}^{n}r_{0}(t=0)c_{\mathrm{eff}}$. Let $r_{0}(t=0)$=$1$, since $r\left( t=0 \right)=0$, we have

$r(t)\simeq{k_{1}}^{n}c_{\mathrm{eff}}t$. (41)

Substituting Eq. 41 into the third equation of Eq. 40 gives

$\dot{r^{*}}+k_{-2}r^{*}\simeq{k_{1}}^{n}k_{2}r_{\mathrm{total}}c_{\mathrm{eff}}t$ (42)

Eq. 42 is linear and can be solved by standard methods. The solution is given below

$r^{*}(t)\simeq{k_{1}}^{n}k_{2}c_{\mathrm{eff}}\left( \frac{e^{-k_{-2}t}-1}{{k_{-2}}^{2}}+\frac{t}{k_{-2}} \right)$

$\simeq{k_{1}}^{n}k_{2}c_{\mathrm{eff}}\left( \frac{1-k_{-2}t+\frac{1}{2}\left( k_{-2}t \right)^{2}-1}{{k_{-2}}^{2}}+\frac{t}{k_{-2}} \right)$

$=k_{\mathrm{eff}}c_{\mathrm{eff}}\frac{t^{2}}{2},$ (43)

where $k_{\mathrm{eff}}={k_{1}}^{n}k_{2}$. As for the steady state activation for stimuli at low concentration Eq. 30, the initial activation depends on an effective binding rate $k_{\mathrm{eff}}$.

For mixture stimulus, as the competition for binding sites for different ligands is negligible when $t$ is small, the binding and activation step is independent for each component. Starting from Eq. 19, it can easily be shown by similar calculation, or by comparison with Eq. 31, that when $t\to0$,

${r_{\mathrm{mix}}}^{*}(t)\simeq k_{\mathrm{eff}}^{\mathrm{mix}}c_{\mathrm{eff}}\frac{t^{2}}{2}$, (44)

where $k_{\mathrm{eff}}^{\mathrm{mix}}=w(n)\sum_{i} k_{\mathrm{eff}}^{i}$. Note that we are still considering the case which each component in the mixture has equal concentration here.

To show that the first-spike latency for mixtures is smaller than the average of its constituent components with the same number of molecules if $n\leq1,$ we consider the mixtures with concentration $c_{0}$ for each component, and the single odorants with concentration $Nc_{0}$. Assuming $n\leq1$, we have

$\left\langle r^{*}\left( c=Nc_{0},t \right) \right\rangle$

$=\left\langle k_{\mathrm{eff}}N^{n}{c_{0}}_{\mathrm{eff}}\frac{t^{2}}{2} \right\rangle$

$=N^{n} \frac{\sum_{i} k_{\mathrm{eff}}^{i}c_{\mathrm{eff}}\frac{t^{2}}{2}}{N}$

$=N^{n-1} \frac{{r_{\mathrm{mix}}}^{*}\left( c=c_{0} \right)}{w(n)}$

$\leq{r_{\mathrm{mix}}}^{*}\left( c=c_{0},t \right)$ (by Eq. 37) (45)

It can easily be shown that by Eq. 36, the inequality sign in Eq. 45 flips if $n\geq1$.

Now, let’s assume that the initial receptor activation at a certain time $t$ is related to the time-projected first-spike latency $f(r^{*})$, defined as the reciprocal of the instantaneous firing rate of neurons [1]. If we assume that $f$ is convex and decreasing with $r^{*}$, which widely holds biologically [2], then by Jensen’s inequality and Eq. 45, we have

$\left\langle f\left( r^{*}\left( c=Nc_{0},t \right) \right) \right\rangle\geq f\left( \left\langle r^{*}\left( c=Nc_{0},t \right) \right\rangle\right)\geq f\left( {r_{\mathrm{mix}}}^{*}\left( c=c_{0},t \right) \right)$ if $n\leq1$ (46)

We may approximate the actual first-spike latency by applying a temporal filter to the time-projected first-spike latency $f(r^{*}(t))$. Since Eq. 46 holds for all time $t$ given it is sufficiently small, this would imply that the first-spike latency for mixtures is smaller than the average of its constituent components with the same number of molecules.

**Reference**

1. Fourcaud-Trocmé N, Brunel N. Dynamics of the instantaneous firing rate in response to changes in input statistics. J Comput Neurosci. 2005;18: 311–321. doi:10.1007/s10827-005-0337-8

2. Thurley K, Senn W, Lüscher H-R. Dopamine increases the gain of the input-output response of rat prefrontal pyramidal neurons. J Neurophysiol. 2008;99: 2985–2997. doi:10.1152/jn.01098.2007
